# Differential processing of small RNAs during endoplasmic reticulum stress

**DOI:** 10.1038/srep46080

**Published:** 2017-04-13

**Authors:** Mikhail V. Mesitov, Ruslan A. Soldatov, Danila M. Zaichenko, Sophie G. Malakho, Tatyana S. Klementyeva, Alisa A. Sokolovskaya, Aslan A. Kubatiev, Andrey A. Mironov, Aleksey A. Moskovtsev

**Affiliations:** 1 Department of Molecular and Cell Pathophysiology, Institute of General Pathology and Pathophysiology, Moscow, Russia; 2 Institute of Information Transmission Problems (Kharkevich Institute) of the Russian Academy of Sciences, Moscow, Russia; 3 Department of Bioengineering and Bioinformatics, Lomonosov Moscow State University, Moscow, Russia; 4 Center of Innovations and Technologies “Biologically Active Compounds and Their Applications,” Russian Academy of Sciences, Moscow, Russia; 5 Russian Medical Academy of Postdoctoral Education, Moscow, Russia

## Abstract

The accumulation of misfolded proteins in the endoplasmic reticulum (ER) lumen due to the disruption of the homeostatic system of the ER leads to the induction of the ER stress response. Cellular stress-induced pathways globally transform genes expression on both the transcriptional and post-transcriptional levels with small RNA involvement as regulators of the stress response. The modulation of small RNA processing might represent an additional layer of a complex stress response program. However, it is poorly understood. Here, we studied changes in expression and small RNAs processing upon ER stress in Jurkat T-cells. Induced by ER-stress, depletion of miRNAs among small RNA composition was accompanied by a global decrease of 3′ mono-adenylated, mono-cytodinylated and a global increase of 3′ mono-uridinylated miRNA isoforms. We observed the specific subset of differentially expressed microRNAs, and also the dramatic induction of 32-nt tRNA fragments precisely phased to 5′ and 3′ ends of tRNA from a subset of tRNA isotypes. The induction of these tRNA fragments was linked to Angiogenin RNase, which mediates translation inhibition. Overall, the global perturbations of the expression and processing of miRNAs and tiRNAs were the most prominent features of small RNA transcriptome changes upon ER stress.

With its protein maturation machinery and other energy-intensive processes, the endoplasmic reticulum (ER) is highly sensitive to numerous factors, such as toxins, excessive protein influx, nutrition and energy deprivation, redox imbalance, and the depletion of Ca^2+^ storage. These factors can cause the accumulation of unfolded/misfolded proteins in the ER lumen, a condition known as ER stress^1^,^2^,^3^. When the threshold level of these damaged and non-native macromolecules is reached, eukaryotic cells activate a cellular reaction called the Unfolded Protein Response (UPR). UPR of high eukaryotes includes three major signaling pathways mediated by three ER transmembrane sensors: protein kinase RNA (PKR)-like ER kinase (PERK), activating transcription factor-6 (ATF6) and inositol-requiring protein-1 (IRE1)^4^. The activation of these sensors results in the production of b-Zip transcription factors, which transduce information to the nucleus and activate the expression of numerous genes involved in protein folding and degradation, amino acid metabolism, redox homeostasis and apoptosis^3^. This complex adaptive program triggers the transcriptome remodeling to restrict the proteotoxicity and compensate for the perturbation of cell homeostasis.

In all eukaryotic cells, the stress conditions lead to adjustments of the cellular gene expression machinery, including a decrease in common transcription, the inhibition of splicing and mRNA export to the cytoplasm, and the temporary attenuation of cap-dependent translation^5^,^6^,^7^,^8^. The ongoing stress-induced cellular processes bring to the fore the post-transcriptional mechanisms of gene expression regulation that cause significant RNome remodeling.

Generally, there are several known post-transcriptional mechanisms of gene expression regulation that cause RNome remodeling. The regulation of the decay of A/U-rich element (ARE)-containing mRNAs (AMD) by the turnover and translation regulatory (TTR) mRNA-binding proteins (RBPs)^9^ and nonsense-mediated decay (NMD)^10^ are among them. TTR proteins play an important role in the cellular stress response through the stabilization of several stress-related ARE-containing mRNAs, such as hsp70^5^.

Another important post-transcriptional regulator involved in transcriptome remodeling is microRNA^11^,^12^,^13^,^14^, which also has a significant impact on different pathological processes. It has been shown that the activity of several classes of small RNAs is tightly regulated in part through their post-transcriptional modifications and alternative processing^15^. The extension of miRNA by non-templated tailing of the 3′ end results in the production of numerous isoforms, so-called “isomiRs”^16^. Diverse post-transcriptional modifications of miRNAs, such as nucleotide additions, have distinct functional consequences^17^,^18^.

Recently, the involvement of miRNAs in the stress responses in metazoa^19^,^20^ and cells^20^,^21^,^22^,^23^ has become a topic of active research. From an evolutionary point of view, as a compartmentalized and relatively late subcellular process, ER stress is of particular interest regarding the role of miRNAs in its regulation.

MiRNAs play an important role in the ER stress response^24^. Some miRNAs recently have been shown to directly regulate the main components of UPR signaling^24^,^25^,^26^. For example, miR-181a-5p, miR-199a-5p and the members of miR-30 family suppress the expression of the ER chaperone BiP (HSPA5, GRP78), one of the main regulators of UPR signaling. Endoribonuclease IRE1α cleaves the precursors of anti-apoptotic miR-17-5p, miR-34a-5p, miR-96-5p and miR-125b-5p, which negatively regulate the expression of CASP2 and TXNIP^27^,^28^. The activation of kinase PERK induces the expression of miR-30c-2-3p, which downregulates the key transcriptional factor XBP1, thereby forming a negative crosstalk between the PERK and IRE1α branches of UPR, in which sustained PERK activation diminishes the pro-survival effect of IRE1α-XBP1 signaling^29^. However, little is known about the differential processing of small RNA transcriptomes during the ER stress response.

Here, we investigated the differential processing and expression of small RNAs in response to ER stress. We induced the acute ER stress response in T-lymphoblastoid Jurkat cells. Throughout the small RNA fraction, we noted the most significant quantitative and qualitative changes in miRNA and tRNA-mapped reads. Using next generation sequencing and bioinformatic analysis, we assessed the differential regulation of miRNAs upon ER stress by the analysis of the mature canonical forms of miRNA and their isoforms. We observed a decrease in the quantity of miRNA-mapped reads and an enormous increase in the Angiogenin-cleaved tRNA fragments (tiRNAs) of the five tRNA isotypes after ER stress induction. We associated an increased level of tiRNAs with their potentially important role in ER stress.

We found that ER stress alters the post-transcriptional modification patterns of mature miRNA 3′ ends in Jurkat T-cells. In particular, we found that the fraction of mono-cytidylation decreases under ER stress and is negatively correlated with the miRNA level. In line with this, C residues are predominantly added to shorter transcripts relative to canonical mature miRNA transcripts. Collectively, our findings reveal tiRNA upregulation, the differential regulation of miRNA expression and a fine-tuned layer of post-transcriptional miRNA modifications as the most significant events in small RNome remodeling during ER stress.

## Results

### Functional annotation of transcriptome in DTT-treated Jurkat cells

We used Jurkat pre-T-cells as the representative model of non-secretory cell type with a low rate of extracellular protein synthesis to dissect ER stress influence on small RNA processing. We induced ER stress in Jurkat T-cells with dithiothreitol (DTT), which reduces the protein disulfide bonds and causes the accumulation of misfolded proteins in the ER lumen^30^. For more detailed assessment of DTT effects on cells, we performed Affymetrix whole transcriptome gene expression analysis with subsequent functional annotation of the classes of genes that are enriched among the genes differentially regulated in DTT-treated cells using Gene Set Enrichment Analysis (GSEA)^31^.

GSEA revealed 35 gene sets upregulated at FDR < 25% (Table S2); 15 gene sets were downregulated at FDR < 25% (Table S3). Among the most significant upregulated gene sets we observed a number of processes directly associated with ER stress (REACTOME UNFOLDED PROTEIN RESPONSE, REACTOME ACTIVATION OF CHAPERONE GENES BY XBP1S, REACTOME PERK REGULATED GENE EXPRESSION, REACTOME ACTIVATION OF GENES BY ATF4), involved in protein folding and glycosylation in ER (REACTOME ASPARAGINE N-LINKED GLYCOSYLATION, REACTOME BIOSYNTHESIS OF THE N-GLYCAN PRECURSOR DOLICHOL LIPID LINKED OLIGOSACCHARIDE LLO AND TRANSFER TO A NASCENT PROTEIN) and probably associated with perturbed vesicular transport (KEGG SNARE INTERACTIONS IN VESICULAR TRANSPORT). Among the downregulated processes we observed a subset of the aforementioned ER-stress affected processes of mRNA processing, splicing and transport, and also REACTOME MICRORNA MIRNA BIOGENESIS. The Affymetrix data were confirmed by quantitative polymerase chain reaction (qPCR). As expected, the expression of the ER stress markers BiP, CHOP and XBP1(S) were increased in Jurkat cells after 6 h of 2.5 mM DTT treatment (Fig. S1A–C). The upregulation of BiP protein level was confirmed by western-blot (Fig. S1D). Based on the expression of ER-stress-specific gene sets and markers, we can suggest ER stress as one of the dominant processes in DTT-treated Jurkat cells.

### ER stress-associated small RNA libraries

To determine differential expression of miRNAs during the ER stress response and to assess the complexity of the small RNA transcriptome in Jurkat T-cells, we performed deep sequencing of small RNAs (sRNAs) up to 35 nucleotides in DTT-treated and non-treated cells from two series of independent biological replicates. Altogether, more than 48 million raw reads of sRNAs were sequenced from 4 samples. After quality control, adaptor clipping and filtering sequences less than 15 nt (see methods for details), we obtained at least 7 million reads per library.

**Figure 1:**
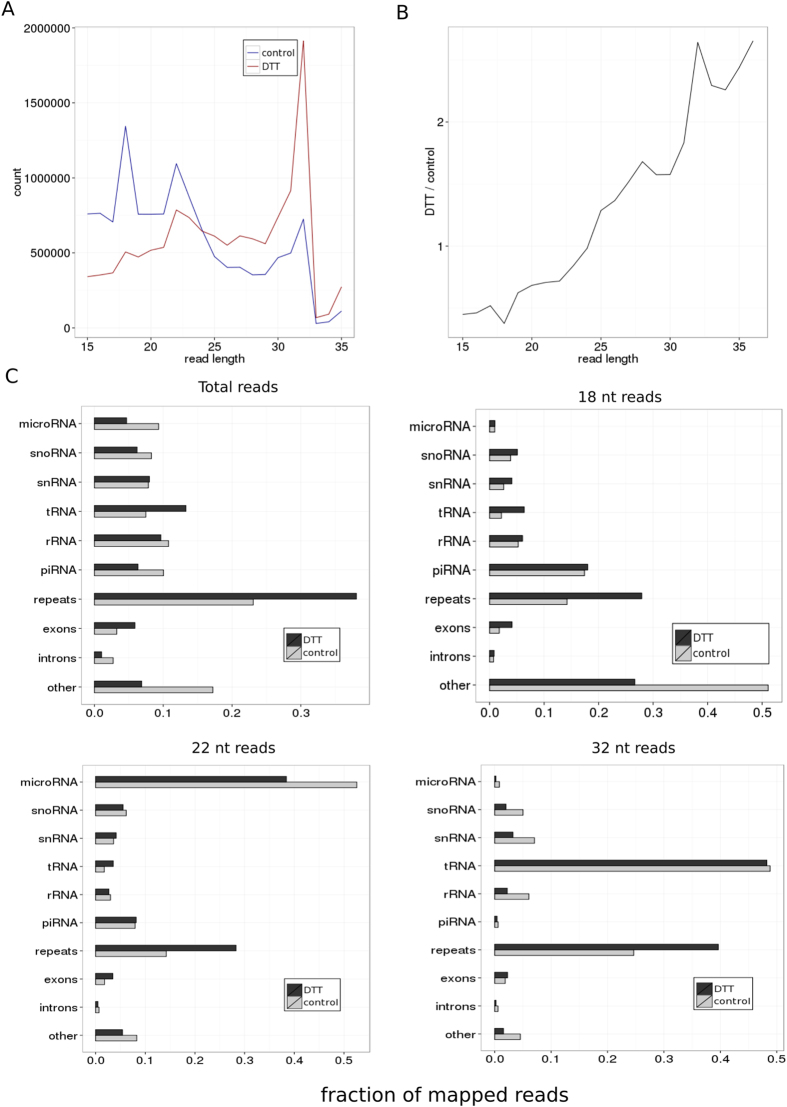
Characterization of small RNA library composition in ER-stressed and control Jurkat T-cells. (**A**) The length distribution of the sequenced reads mapped without mismatches. (**B**) The ratio of abundance of the sequenced reads in ER-stressed and control Jurkat T-cells. (**C**) The distribution of reads for different RNA classes. The distributions are obtained for the total mapped reads and separately for reads 18, 22 and 32 nucleotides in length.

The length distribution of small RNAs revealed three peaks corresponding to 18 nt, 22 nt and 32 nt (Fig. 1A) and an overall increase of read length in response to ER stress (Fig. 1B). To characterize the sRNA composition, we mapped sequenced reads to annotated small non-coding RNAs, protein-coding genes, and repeats (Fig. 1C). The read distributions of miRNAs and tRNA-derived fragments showed single peaks at 22 nt and 32 nt, indicating the non-random processing of these species, whereas other annotated RNA species showed a random length distribution and relatively low levels of their degradation products (Fig. S2). In line with this, the 22-nt and 32-nt reads were dominated by miRNAs and tRNA-derived fragments, respectively (Fig. 1C). In contrast, we didn’t identify the specific class of RNAs that might be linked to production of 18-nt reads (Fig. 1C, Fig. S2). We thus focused further analysis on microRNAs and tRNA-derived fragments. Interestingly, these small RNA species demonstrated opposite trends: microRNAs were significantly downregulated, and tRNA-derived fragments were strongly upregulated under ER stress conditions compared to other small RNA species (Fig. 1B,C).

### ER stress-associated tRNA cleavage

**Figure 2:**
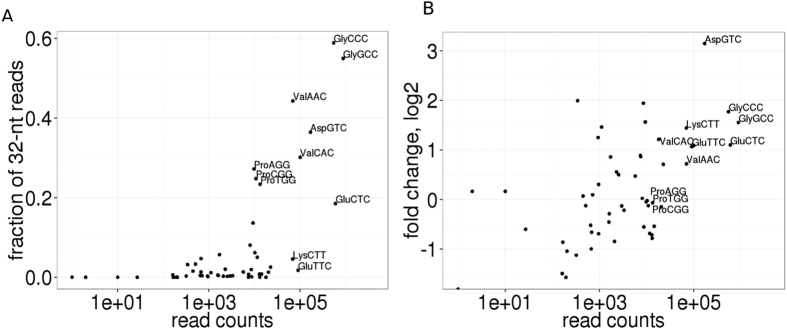
tRNA-derived fragments exhibit unique features. (**A**) Scatter plot showing the fraction of 32-nt reads among total counts versus total counts for tRNA reads. Total counts are summed among the control and stressed conditions. (**B**) Scatter plot showing the ratio of reads normalized by the median ratio of tRNA isotypes in ER-stressed cells compared to control cells (logFC) on the dependence of total counts for tRNA reads.

**Figure 3:**
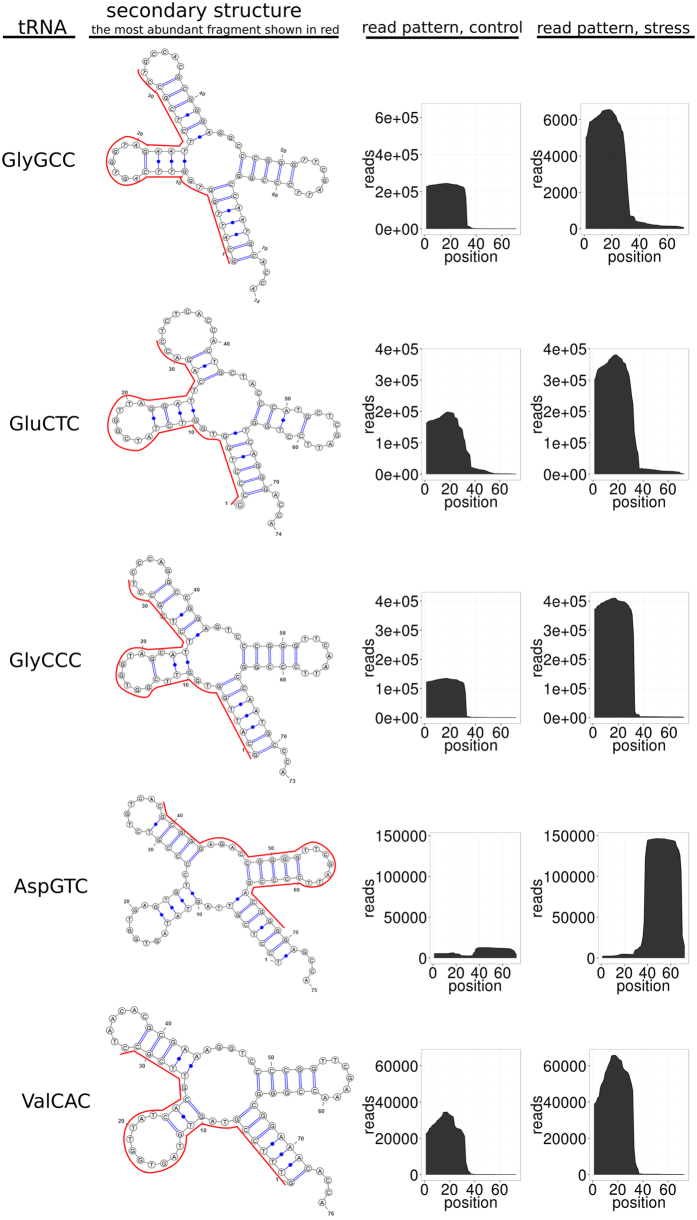
Secondary structures, the most abundant fragments (red) and read patterns of the five most abundant tRNA isotypes in 32-nt read fractions in ER-stressed and control Jurkat T-cells.

The relative quantity of tRNA-derived fragments was increased by 1.8-fold, from 718,000 reads (7.5% of total reads) in control cells to 1,480,000 reads (13% of total reads) under ER stress induction (Fig. 1C, Fig. S2). We found that tRNA isotypes with a high quantity of reads contained a large fraction of 32-nt reads among their sequenced fragments (Fig. 2A). The five tRNA isotypes that gave rise to almost 76% and 86% of all 32nt RNA fragments in ER-stressed and control cells, respectively, included glycine (Gly-tRNA^GCC^ and Gly-tRNA^CCC^), glutamic acid (Glu-tRNA^CTC^), aspartic acid (Asp-tRNA^GTC^) and valine (Val-tRNA^CAC^). The total number of reads of these tRNA species significantly increased in response to ER stress compared to other tRNA species (Fig. 2B). The vast majority of 32-nt fragments produced from these tRNAs were precisely phased 5′ halves with the cleavage site within the anticodon loop (Fig. 3). The exception was Asp-tRNA^GTC^-derived fragments, which are the only dominant 32-nt fragments to be processed from 3′ halves of the tRNA (Fig. 3).

**Figure 4:**
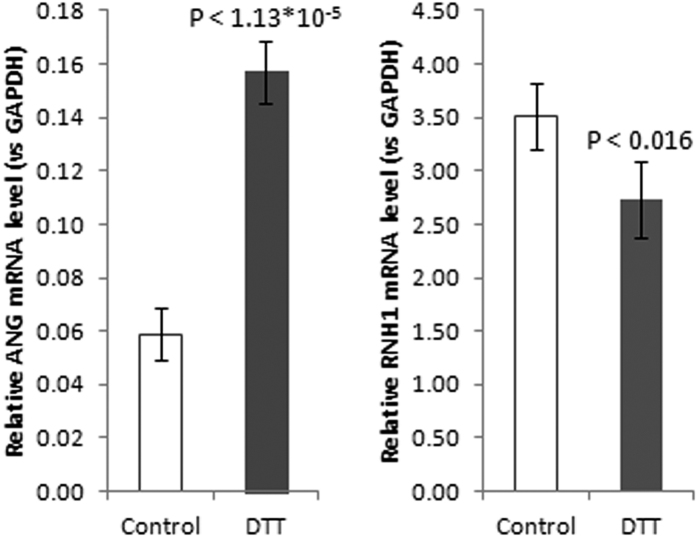
Expression levels of Angiogenin (ANG) and its inhibitor RNH1 mRNAs in Jurkat T-cells under ER stress. Data are presented as the mean ± SD (n = 4). The P value was calculated versus controls by two-tailed Student’s t-test.

The characteristic patterns of tRNA-derived fragments resembled those generated by endoribonuclease A Angiogenin (ANG), which cleaves tRNA molecules within the anticodon loop^32^,^33^. The accumulation of tRNA fragments is often associated with stress conditions^34^,^35^. Thus, we tested whether observed generation of tRNA fragments is accompanied by variations in the expression levels of ANG and its inhibitor ribonuclease/angiogenin inhibitor 1 (RNH1). Under stress conditions, the dissociation of RNH1 from ANG in cytoplasm promotes its ribonucleolytic activity^36^. Indeed, the expression of *Ang* mRNA was increased in response to ER stress, whereas the expression of *Rnh1* mRNA was moderately but significantly downregulated under the same conditions (Fig. 4). Such tRNA-derived small RNA species, so-called tiRNAs, were shown to induce translational attenuation, interfering with the eIF4F complex, thereby contributing to the suppression of global protein synthesis along with the phosphorylation of eIF2α^32^,^33^,^37^.

To check the independence of these effects from cell line and inductor specificity, we conducted similar analyses of RNH1 and ANG differential expression using an alternative cell line (EA.hy926) and additional treatment (tunicamycin 10 μg/ml). As a result, in both cell lines and under both treatment we obtained the generally similar patterns of ANG expression, which were less prominent in Jurkat cells under tunicamycin treatment (Fig. S3B), but robust in adherent endothelial cells EA.hy 926 (Figs S4B and S5B). RNH1 downregulation was less robust (Figs S3C and S5C) with the exception of DTT treatment of EA.hy 926 cells (Fig. S4C).

### miRNome and differentially expressed miRNAs in Jurkat cells upon ER stress

**Figure 5:**
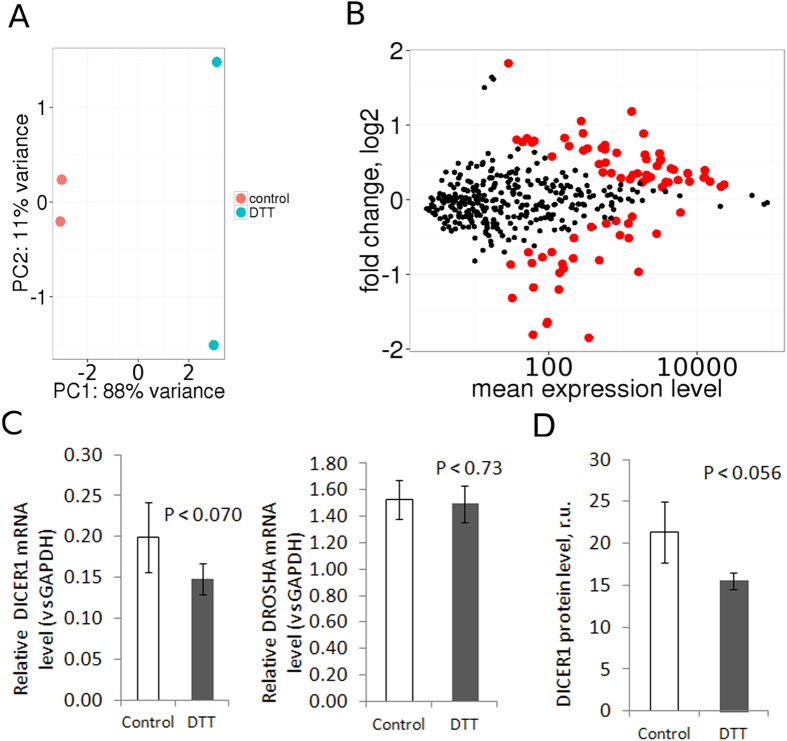
Differential expression analysis of miRNAs in Jurkat T-cells under ER stress. (**A**) Principle component analysis of miRNA expression among samples distinguishes the control and ER stress conditions (**B**) Fold change versus the expression level of differentially expressed miRNAs with FDR < 0.05 are depicted in red (**C**) Expression levels of *Dicer1* and *Drosha* mRNAs do not change significantly after 6 hours of ER stress induction in Jurkat T-cells (n = 5). (**D**) Expression level of DICER1 protein in Jurkat cells under DTT 2.5 mM treatment, 6 h (n = 3). Data are presented as the mean ± SD. The P value was calculated versus controls by two-tailed Student’s t-test.

We characterized the mature miRNome in Jurkat cells by mapping reads using Bowtie^38^ with perfect matching against miRBase v.21^39^ and identified 451 miRNAs. To determine the miRNA candidates implicated in ER stress, we performed differential expression analysis using DESeq2^40^ (details in Methods). Principal component analysis based on miRNA expression separated ER-stressed from control samples (Fig. 5A).

Because the Jurkat cell line expresses T-cell characteristics and it was derived from a patient with acute T-lymphoblastic leukemia^41^, we expected to find a T-cell precursor-specific miRNA expression pattern. One of the highly expressed miRNAs in Jurkat cells was miR-181a (miR-181a-5p, 54705.6 RPM), which was previously characterized as an important regulator of thymocyte-positive selection by the repression of Bcl-2, CD69, and T-cell receptors^42^ in the early stages of T-cell maturation^43^. Interestingly, among highly abundant miR-181a-1-5p, miR-181a-1-3p, and miR-181a-2-5p, only miR-181a-1-3p (miR-181a*) expression was significantly decreased upon ER stress in Jurkat cells.

Highly expressed miR-191-5p in Jurkat cells (20616.4 RPM) was shown earlier as stress-inducible miRNA in response to X-ray treatment in a human TK6 lymphoblast cell line^44^. Furthermore, miR-191-overexpressing breast cancer cells showed increased survival in response to chemotherapeutic drug or hypoxia treatment^45^. However, we did not detect its significant expression changes upon ER stress.

The most enriched miRNAs in Jurkat cells were miR-92a-1-3p (88468.7 RPM) and miR-92a-2-3p (80318.7 RPM) that belongs to OncomiR-1 (miR-17~92 cluster). We also did not detect any changes in their expression upon ER stress but did for other members of the miR-17~92 cluster, such as miR-92a-1-5p, which was the most significantly downregulated miRNA with −1.95 LFC (348.0 RPM). At the medium level, we also detected miRNAs belonging to the two miR-17~92 homologous clusters - miR-106a~363 and miR-106b~25: miR-106a-5p (698.7 RPM), miR-363-3p (749.2 RPM), miR-106b-5p (251.3 RPM), miR-25-3p (3397.2 RPM). The miR-17~92 cluster is highly expressed in lymphocytes and promotes the survival of early B cell progenitors^46^. The important targets of the miR-17~92 cluster are the pro-apoptotic protein Bim and tumor suppressor Pten^47^, and this cluster is predominantly pro-survival.

Another cluster whose members were highly enriched was the miR-15a/miR-16-1 (miR-15/16) cluster. We noticed a significant upregulation of miR-16-1-5p (12706.6 RPM), miR-16-2-5p (12713.1 RPM), and miR-15a-5p (316.7 RPM). The direct target of the miR-16 family is Cyclin D1 (CCND1); moreover, the expression levels of some other cell cycle regulators, such as Cyclin D3 (CCND3), Cyclin E1 (CCNE1) and CDK6, are under miR-16 control^48^. The upregulation of miR-16 was also shown during UV-induced cellular stress, where it represses the expression of the checkpoint gene CDC25a and regulates cell proliferation^49^. Because cell cycle changes represent the prominent cell stress-related event we suggest miR-15/16 as stress-associated miRNAs, which induction is common for different cellular stress responses.

Interestingly, we observed highly expressed (7498.4 RPM) and reliably upregulated miR-30d-5p, which, along with the above-mentioned highly enriched miR-181a-1-5p and miR-181a-2-5p together with miR-199a-5p (not detected in Jurkat cells), has BiP as the validated target^50^.

Overall, among 451 identified miRNAs in Jurkat cells, we observed the differential expression of 105 miRNAs with FDR < 0.05 (Fig. 5B). Of these, 69 miRNAs were upregulated, and 36 miRNAs were downregulated in response to ER stress. Importantly, among the group of upregulated miRNAs, nine were previously described as ER-stress responsive: miR-17-5p^28^, miR-30e-5p, miR-30c-5p, miR-30d-5p^51^, miR-221-3p, miR-222-3p^52^, miR-23a-3p^53^, miR-25-3p^54^, and miR-140-5p^55^ (Supplement 2). The majority of these miRNAs are associated with cell death, and only miR-17-5p and miR-25-3p play pro-adaptive roles^28^,^54^. However, the upregulation of all of these miRNAs was moderate and did not exceed 2-fold compared to control. Notably, validated ER stress-associated miRNAs did not occur amongst downregulated miRNAs. The majority of highly enriched miRNAs did not change their expression under ER stress conditions.

At the global level, we observed a decrease in the quantity of miRNA reads compared to other sRNA classes with 9.0% reads (989k reads) perfectly aligned to miRNA loci in control cells compared to 4.7% (521k reads) in ER-stressed cells (Fig. 1C). Considering the identification by GSEA of a downregulated miRNA biogenesis gene set, we wondered whether the ER stress somehow causes global miRNA downregulation so that the expression ranking was not affected. It is well known that the production of mature miRNAs strongly depends on the modulation of endoribonuclease III DICERI expression and expression of the main microprocessor component DROSHA^56^. Therefore, we examined the expression levels of these important miRNA biogenesis factors in control and ER-stressed cells. We observed that the expression levels of DICER1 mRNA and Drosha mRNA were not significantly different among samples (Fig. 5C).

We also checked DICER1 protein level quantitatively under DTT-induced ER stress in Jurkat cells using ELISA (Fig. 5D). We observed DICER1 protein level downregulation which was stronger than a change in DICER1 mRNA level. To check the robustness of these effects, we performed RT-qPCR and ELISA analysis using tunicamycin treatment and EA.hy926 cell line. In tunicamycin-treated Jurkat cells and both DTT- and tunicamycin-treated EA.hy926 cell line, we observed significant downregulation of DICER1 mRNA (Figs S6A and S8A,C). DICER1 protein level was downregulated in both treatments of EA.hy926 cell line (Fig. S9) but unexpectedly upregulated in Jurkat cell line under tunicamycin treatment comparing with DMSO-treated control cells (Fig. S7B). In contrast to DICER1, we did not notice any tendency in DROSHA mRNA differential expression under tunicamycin treatment in both cell lines (Figs S6B and S8D) excluding significant downregulation in DTT-treated EA.hy926 cells (Fig. S8B) Thus, the decrease in the abundance of miRNA-mapped reads could be related to ER stress-induced miRNA biogenesis downregulation.

### Expression of isomiRs in Jurkat T-cells

**Figure 6:**
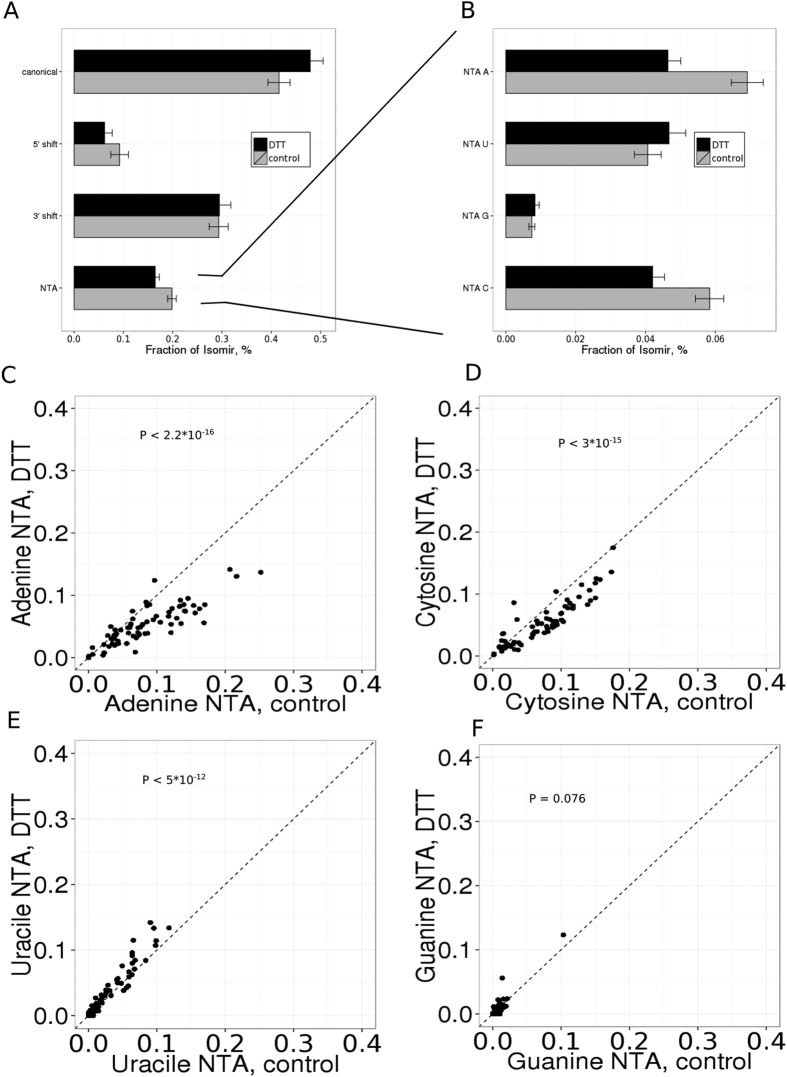
The 3′ end posttranscriptional modifications exhibit global changes upon ER stress. (**A**) Mean fraction of canonical, 5′ shifted, 3′ shifted and 3′ end modified (NTA) isomiRs among abundant miRNAs in ER-stressed and control cells. (**B**) Mean fraction of different types of 3′ NTAs in ER-stressed and control cells. (**C**–**F**) Comparison of 3′ NTA isomiR fractions in ER-stressed and control cells for each miRNA. Only miRNAs with at least 500 mapped reads were considered.

Facing a proven regulatory impact and widespread functional consequences of isomiRs expression patterns^16^,^57^,^58^,^59^,^60^, we focused on non-templated 3′ nucleotide additions (3′ NTA) and shifts of the 5′ and 3′ ends of mature miRNAs. We observed that more than half of the miRNA loci gave rise to isomiRs that differed from the canonical one annotated in miRBase v.21 (Fig. 6A). IsomiRs with the shift of the 5′ end are relatively depleted (Fig. 6A) as expected from a distinct seed sequence leading to a new pool of targeted genes^61^. The most dominant isomiR type is a shift of the 3′ end of mature miRNAs in line with its moderate functional consequences.

### miRNA non-templated 3′ nucleotide additions

Given the regulatory potential of 33′ NTAs, such as the modulation of miRNA stability^58^,^62^, the repressive activity towards target mRNAs^63^, localization^64^ and association with AGO proteins^65^, we next asked whether ER stress, a strong pathological stimulus, induces such variations, which could modulate the miRNA transcriptome and subsequently miRNA-mediated control of numerous RNA targets. We examined 33′ NTAs up to three additional residues (see details in Methods). Approximately 20% of miRNA reads contain 33′ NTA, which decreases up to 16% upon stress induction. The overwhelming majority of 33′ NTA was represented by the single nucleotide additions, with only 3.5% of DTT-treated and 4.1% of control samples of 33′ NTA corresponded to two and three nucleotide additions, respectively. Among highly expressed miRNAs, 35% of reads of miR-26a-5p, 44% of reads of miR-130b-3p and 47% of reads of miR-148b-3p possessed a single 33′ NTA. A, U and C additions were the most common types of 33′ NTA, whereas the addition of G occurred much more rarely (Fig. 6B), consistent with observations in previous studies^66^,^67^.

We found that the total mono-adenylation ratio is reduced from 8.4% to 5.6% in Jurkat T-cells under ER stress (P < 2.2 * 10^−16^, Fig. 6C). In contrast, the mono-uridylation ratio is slightly increased from 5.2% to 6.5% (P < 5 * 10^−12^, Fig. 6E). We also observed a moderate but statistically significant reduction in mono-cytidylation from 6.6% to 5.0% in response to ER stress (P < 3 * 10^−15^, Fig. 6D). There are no correlations between the fold change in miRNA expression and the variation of the NTA fraction, indicating that the variation of 33′ NTA is not specific to the regulation of the subset of microRNAs (Fig. S10). The addition of adenine to the 3′ end of miRNA usually associates with destabilization of mature miRNA^58^,^62^,^68^,^69^, whereas the uridylation of the 3′ end reflects either the degradation or stabilization signal, depending on the quantity of added U residues and the stage of miRNA biogenesis^63^,^70^,^71^. The role and mechanism of 3′ C additions remain largely unknown. Interestingly, a reduction in mono-cytidylation ratio upon ER stress negatively correlates with miRNA expression in contrast to other 3′ NTA types (Fig. S10). These results provide direct evidence that ER stress alters the miRNA 3′ end nucleotide modification patterns.

Mature miRNA can be derived from both the 5p and 3p arms of the hairpin precursor and be modified by 3′ NTA independently. The 3′ NTAs of the 5p arm occur after DICER1 cleavage in mature miRNA, whereas these modifications of the 3p arm can also occur before DICER1 cleavage in pre-miRNA^72^. The comparison of modification rates of 5p and 3p miRNA arms assists in the identification of the processing step for the specific types of 3′ NTAs. It has been reported previously that the mono-uridylation of a single nucleotide 3′-overhang of type II pre-miRNAs belonged to the let-7 family apparently facilitates pre-miRNA export by Exportin 5 and DICER1 processing^73^. In this case, 3p-miRNA is already modified with 3′ U-addition before DICER1 cleavage, whereas 5p-miRNA can be modified by 3′ NTAs afterward. In line with the above mechanism, the mono-uridylation of 3p-miRNAs occurs 2.7 times more often compared to 5p-miRNAs in Jurkat T-cells (Fig. S11). In contrast, there is opposite bias towards the 5′ miRNA arm for mono-cytidylation (Fig. S11). The mono-uridylation of 3p-miRNAs could reflect either the unfinished marking for degradation^66^,^70^,^74^ or the promotion of type II pre-miRNA to mature miRNA processing^73^. The mono-uridylation of 5p-miRNA does not lead to an alteration of its stability, as was mentioned previously^71^. Accordingly, despite the slight increase of miRNA 3′ end mono-uridylation, the induction of ER-stress in Jurkat cells does not influence non-templated 3′ single U addition to miRNA precursors.

### Variation of miRNA length during ER stress

Because the length distribution of miRNA reads reflects transcriptome remodeling due to diverse processes, such as stabilization, tailing and trimming with subsequent degradation^68^,^69^,^75^, we decided to analyze the global patterns of miRNA length variation in response to ER stress in Jurkat T-cells. We asked whether the 3′ NTAs are correlated with the isomiR length distribution in a normal state and under ER stress. We observed an overall increase of miRNA length after ER stress induction (Fig. S12A, p < 2.2 * 10^−16^). The comparison of the length distributions of isomiR reads with 3′ NTA and other miRNA reads without 3′ NTA demonstrated heterogeneous length preferences for pre-modified miRNAs (Fig. S12B–F). Uracil and adenine are preferentially added to 22 nt pre-modified miRNAs, while cytosine is preferentially added to 21 nt pre-modified miRNAs, indicating their distinct mechanisms of origin (Fig. S12C–F). We observed a profound shift toward 22-nt pre-modified miRNA species of adenine NTA after stress induction with 45% and 55% in control and ER-stressed Jurkat T-cells, respectively (Fig. S12C).

### The differential expression of Ago1-4 genes in response to ER stress

**Figure 7:**
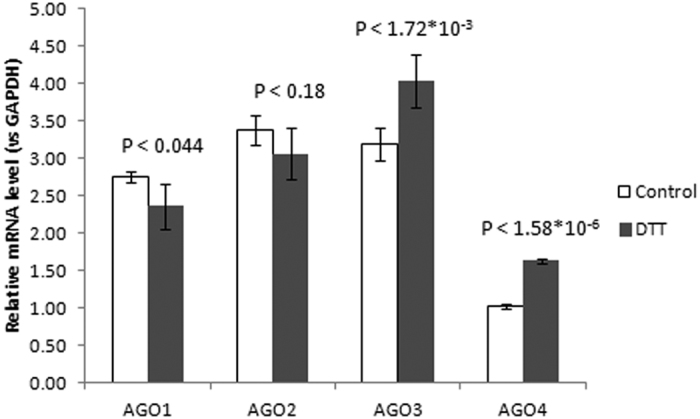
Differential expression of *Ago1-4* during the ER stress response. Average means were obtained from at least 5 independent biological replicates. Data are presented as the mean ± SD (n = 5). The P value was calculated versus controls by two-tailed Student’s t-test.

The type of 33′ NTA and variations of miRNA length could be associated with preferential isomiR distribution among the different AGO proteins. We next asked whether the isomiR distribution between libraries correlated with the expression of *Ago1-4* mRNAs. It has been reported that isomiRs with 3′ mono-A-addition are primarily selected onto AGO1 and AGO2 and less onto AGO3, whereas isomiRs with 3′ mono-U-addition were preferably loaded onto AGO3 and AGO2 and were rather rare in AGO1^65^,^76^. Dueck and colleagues reported that 22-nt miRNAs mostly accumulated in AGO2, whereas 23-nt and 24-nt miRNAs were preferably selected by AGO1 and AGO3^65^. Furthermore, the expression of mature miRNAs and AGO proteins was closely related because the AGOs are the major factors that determine the stability and abundance of miRNAs, which in turn influence AGO levels^77^,^78^. We found that *Ago1* mRNA was moderately downregulated by 0.84-fold, whereas the expression of *Ago2* did not change significantly in response to ER stress (Fig. 7). Conversely, the expression of *Ago3* and *Ago4* mRNAs was moderately increased by 1.22-fold and 1.46-fold, respectively. Together, ER stress induction in Jurkat T-cells promotes the downregulation of *Ago1* accompanied by decreased non-templated A addition and the reduction of isomiR fractions shorter than 22 nt. However, we observed the upregulation of *Ago3* and *Ago4*, which was accompanied by increased U-addition and an increase in isomiR fractions 22nt and longer. The tendency of AGOs mRNAs expression changes was generally maintained in Jurkat cells also under the other ER stress inducer (tunicamycin) treatment, except that of AGO1 mRNA (Fig. S13A). In EA.hy926 cell line under DTT and tunicamycin treatment we observed similar and significant *Ago1,2* mRNAs downregulation but non-robust *Ago3,4* mRNAs changes (Fig. S13B,C). Altogether, these results support the concept that variations in length and 3′ additions of non-templated nucleotides can influence miRNA selection onto different AGO proteins.

## Discussion

Recent evidence suggests the emerging role of miRNAs in the shaping of the endoplasmic reticulum stress response in mammalian cells. To clarify the ER stress-associated changes that occur in the fraction of small RNAs, we performed versatile global small RNA analysis. We found changes in the enrichment of several small RNA classes in response to DTT-induced ER stress, and two of them were considered most significant: a decrease in normalized quantity of reads aligned to miRNA loci and an increase in the tRNA-derived fragments (tiRNAs). Affymetrix whole transcriptome gene expression analysis of ER stressed cells revealed “Reactome miRNA biogenesis” among downregulated processes, from which, interestingly, processes related with RNA turnover were most prevalent. Subsequent RT-qPCR and ELISA of a key component of miRNA biogenesis DICER1 allowed us to suggest ER stress-affected miRNA biogenesis as the cause of observed miRNA-mapped reads downregulation.

As expected, the most enriched of identified 451 mature miRNAs were represented by ones previously found in lymphoblasts but also by cancer-related miRNAs. Further differential expression analysis of miRNAs and their isomiRs revealed a total of 105 differentially regulated miRNAs (FDR < 0.05) Among these, we found nine previously described ER stress-associated miRNAs. Interestingly, the most enriched miRNAs did not significantly change their expression with several exceptions. It should be noted, however, that the list of identified DE miRNAs only partially reflects *bona fide* DE miRNAs due to insufficient detection power^79^. Despite potentially noisy detection of expression changes for particular small RNAs, statistical aggregation among many small RNAs confers robustness to conclusions regarding global effects of ER stress on small RNome.

As part of differential expression analysis, we observed for the first time that commonly expressed in Jurkat T-cells isomiRs undergo a significant change in their pattern upon ER stress. Interestingly, a subset of miRNAs had significant 3′ mono-adenine or mono-uridine additions, related to those previously described as frequently targeted for these additions in mammals^57^. In our work, the mono-adenylation ratio of miRNA 3′ ends decreased, whereas the mono-uridylation ratio moderately increased. Previously, it was reported that in animals, the addition of non-templated adenines could lead to the trimming, destabilization and subsequent decay of miRNAs^58^,^62^,^68^,^69^. In fruit flies, during the maternal-to-zygotic transition, the adenylation of the 3′ end of the maternally inherited miRNA by Wispy promotes its degradation in earlier embryos^58^. The addition of an oligouridine tail to the 3′ end of the pre-miRNAs leads to rapid turnover, whereas mono-uridylation promotes their processing by DICER1^66^,^70^,^73^,^74^. The production of one nucleotide longer isoform of oncogenic miR-21 leads to its adenylation by noncanonical poly(A) polymerase PAPD5 and subsequent degradation through PARN-mediated 3′ to 5′ decay^69^. Enhanced uridylation of 3p-miRNAs apparently reflexes the pre-miRNA modification, whereas significantly lower 5p-miRNA uridylation ratio might be connected with target recognition. However, Thornton JE and colleagues reported that the mono-uridylation of 5p-miRNA does not lead to alterations of its stability, but there is evidence that the addition of uridine residues by Zcchc11 to the 3′ end of mature miR-26 reduces its activity towards the target mRNA of interleukin-6^63^,^71^. The analysis of the isomiR length distribution between control and DTT-treated samples reveals its significant shift towards longer isoforms after ER stress induction. Interestingly, variations of miRNA length could also occur during some physiological processes, such as neuronal development, and are associated with the relative abundance of Argonaute proteins^65^,^75^. We observed that the length of mono-cytidylated isomiRs is shifted toward 21 nt, whereas the length of mono-uridylated and mono-adenylated isomiRs are shifted toward 22 nt. The length distribution of isomiRs bearing non-templated C on the 3′ ends clearly peaks at 21 nt. Moreover, we revealed that the reduction in the mono-cytidylation ratio negatively correlates with miRNA expression. Collectively, these results imply that mono-cytidylation might be a stronger degradation signal than mono-adenylation. The role and mechanism of 3′ C additions remain largely unknown.

Variations in the 5′-end of a mature miRNA can give rise to a new isomiR with different seed region. Such isomiRs can possess a distinct spectrum of targets compared with canonical ones. The functionality of 5′isomiRs has been confirmed *in vivo*^59^. Different 5′ isomiRs that originated from the same locus could be deregulated under different pathological conditions, such as cancer^80^. We also examined the variation of the 5′ miRNA end consisted in template elongation or shortening by one or two nucleotides in Jurkat cells under normal conditions and during ER stress. Overall, our analysis revealed that the miRNAs with non-canonical 5′ isomiRs accounted for 10% of the total identified miRNAs. Of these, miR-140-3p, miR-142-3p and hsa-miR-101-3p (detected separately from mir-101-1 and mir-101-2) were reported previously to have abundant 5′ isomiRs in different human tissues and cell lines^57^,^81^. The 5′isomiR distributions were almost entirely the same for all libraries and did not change during the ER stress response. This finding indicates that significant proportions of these miRNAs’ 5′ isomiRs might be due to natural features determined by the structure of miRNA precursors than the specific modulation of miRNA biogenesis.

We show here that ER stress promotes the generation of 32-nt-long tiRNA-like tRNA fragments in human T-cells. The vast majority of these fragments are the 5′ halves of the corresponding tRNA molecules with the cleavage site within the anticodon loop. However, tiRNA-like fragments of Asp-tRNA^GTC^ are processed from the 3′ halves of the tRNA. Concomitantly, the expression of *Ang* was increased, and the expression of *Rnh* was moderately decreased, which implied that ER stress induced the activation of ANG-mediated tRNA cleavage.

As previously shown, several types of stresses can induce tRNA cleavage within the anticodon loop, resulting in the generation of short tRNA fragments mainly by RNase A Angiogenin (ANG), named tRNA-derived, stress-induced small RNAs (tiRNAs)^32^,^33^. This phenomenon was described for different mammalian cells and tissues^32^. The accumulation of tiRNAs appears to promote phospho-eIF2α-independent translational arrest by displacing the eIF4F complex components from capped or uncapped mRNAs with the mediation of YB-1, and the subsequent assembly of stress granules^33^,^34^,^37^. This could contribute to a decrease in the newly synthesized protein influx into the ER and prevention of energy loss during stress. In line with our observation, recent work has shown that Angiogenin promoted the adaptation to ER stress in kidney injury through the inhibition of the translation and activation of stress granules^82^. We suggest that the Angiogenin-promoted adaptation to ER stress is induced through the generation of tiRNA fragments, which inhibit translation and promote the assembly of stress granules.

Thus, we present evidence that ER stress alters miRNA expression and post-transcriptional modification patterns of miRNAs in human T-cells, resulting in variations of non-templated nucleotide additions. Of these, the adenylation of the 3′ end is a hallmark of potential miRNA degradation and decreases under ER stress, thereby implying the stabilization of mature miRNAs. Additionally, a negative correlation between miRNA expression and the mono-cytidylation ratio suggests that C-addition also might be a signal of miRNA decay, possibly stronger than adenylation. We observed the differential expression of another class of small RNA (tiRNA), which we suggest is an important transcriptionally independent modulator of the ER stress response.

## Material and Methods

### Cell line, culture conditions and treatment

Jurkat cells were obtained from the Russian cell culture collection (Institute of Cytology Russian Academy of Science (ECCO member), Saint Petersburg, Russia) and cultured in RPMI1640 (Gibco) containing 10% fetal bovine serum (Gibco) and gentamicin (50 μg/mL; Gibco) at 37 °C in a humidified incubator at 5% CO2. Human EA.hy926 endothelial cells were kindly provided by Dr. C.J. Edgel (University of North Carolina, USA) and cultured in Dulbecco’s modified Eagle’s medium (Life Technologies) supplemented with 10% (v/v) FBS (Life Technologies), 1% non-essential amino acids, 2 mM glutamine (Paneko), HAT supplement (Life Technologies), and 50 μg/ml gentamicin (Life Technologies) at 37 °C in a humidified atmosphere containing 5% CO2. To induce ER stress, the cells were treated for 6 hours with 2.5 mM Dithiothreitol (Sigma-Aldrich) or 10 μg/ml tunicamycin (Sigma-Aldrich), dissolved in DMSO (Sigma-Aldrich).

### RNA isolation, reverse transcription and qPCR

Total RNA was isolated from Jurkat cells using an RNeasy Mini Kit (Qiagen) followed by DNase I treatment (Thermo Fisher Scientific) according to the manufacturer’s protocols. One microgram of each RNA sample was reverse-transcribed using random hexamer primers with a RevertAid H Minus First Strand cDNA Synthesis Kit (Thermo Fisher Scientific) following the manufacturer’s instructions. The resulting cDNA was used for qRT-PCR, which was performed using a CFX96 Real-Time PCR System (Bio-Rad Laboratories) and a Maxima SYBR Green/ROX qPCR Master Mix (Thermo Fisher Scientific). The primers used are listed in Table S1. The relative expression of transcripts was normalized to that of the GAPDH reference internal control mRNA from each sample using the 2^−ΔCt^ method. P-values were calculated using two-tailed unpaired Student’s t-test.

### Western blot and ELISA

The BiP protein level was determined by western blot as previously described^83^. In brief, cells after treatment were washed with PBS twice and were lysed with RIPA buffer (50 mM Tris, 150 mM NaCl, 0.5% Sodium deoxycholic acid, 1% (v/v) NP-40, 0.1% (w/v) SDS). For total protein concentration determination Bradford method and spectrophotometer NanoDrop (Thermo Scientific) were used. 20 μg of total protein per sample was used for 10% SDS-PAGE electrophoresis and semi-dry transfer to PVDF membrane. After that membranes were blocked and incubated with primary antibodies overnight (Abcam). After washing, membranes were incubated with secondary HRP-conjugated antibodies with subsequent detection using Kodak 440CF Image station and Amersham ECL Western Blotting Detection Kit (GE Healthcare Life Sciences) according to manufacturer’s instructions. For DICER1 protein level measurement ELISA was used. In brief, cells after treatment were washed with PBS two times and were lysed with freeze-faw method with subsequent total protein concentration determination with Qubit Protein Assay Kit (Life Technologies) and Qubit Fluorometer (Invitrogen). Sandwich enzyme immunoassay was performed according to the manufacturer’s protocols (Cloud-Clone Corp.) Absorbance was measured at a wavelength of 450 nm using multimodal microplate reader Chameleon (Hidex). The concentration of Dicer 1 in the samples was then determined by comparing the O.D. of the samples to the standard curve.

### Deep sequencing of small RNAs

Small RNAs (sRNAs) were extracted from Jurkat cells using a mirVana miRNA Isolation Kit (Ambion) according to the manufacturer’s protocols. An additional selection of the entire sRNA fraction between 15–30 nt was performed by isolation from 15% denaturing PAGE and subsequent ethanol precipitation. sRNA sequencing libraries were prepared using Illumina TruSeq small RNA library preparation kit (Illumina) following the manufacturer’s instructions. In brief, 0.3-0.5 μg of sRNA fraction was ligated sequentially at the 3′ and 5′ end with synthetic RNA adapter, reverse transcribed and enriched in PCR with Illumina sequencing primers with barcodes. All libraries before the enrichment PCR were normalized by using qPCR with SYBR Green and with primers identical to Illumina TruSeq PCR primers but without end modifications. The number of cycles of subsequent enrichment PCR was determined based on the threshold cycle and was one cycle less than the threshold cycle. The amplified libraries were subsequently purified by 10% PAGE according to the expected product size. To validate the library efficiency and quantification, qPCR was carried out with SYBR Green Assays according to Illumina qPCR Library quantification protocol (Illumina). Normalized small RNA libraries were sequenced for 36 cycles, of three in a separate lane, using Illumina GAIIx Genome Analyzer (Illumina).

### Computational analysis of small RNAs

We removed low-quality ends and clipped the 3′ adaptor from raw reads using Cutadapt^84^ followed by filtering reads longer than 14 nt. Control and experimental samples contained 15.4 and 19.7 million processed reads, respectively. To characterize the composition of small RNA species, reads were mapped by Bowtie^38^ with perfect matching against miRBase v.21^39^, tRNAdb^85^, piRBase^86^, tracks of rRNA, snoRNA, snRNA, and protein-coding genes downloaded from Ensemble release 84^87^ and a repeatmasker track downloaded from the UCSC genome browser^87^. For the analysis of tRNA fragments, unambiguously mapped reads were excluded. IsomiRs were defined as reads that have 5′ end less than 3 nt apart from the 5′ end of mature microRNA, at most 3 non-template nucleotides at the 3′ end and without mismatches at internal nucleotides. Mature microRNAs from 5′ and 3′ arms of pre-microRNA were analyzed separately. IsomiRs were mapped according to the previously described procedure^88^. Read counts of mature microRNAs from the 5′ and 3′ arms of pre-microRNA were calculated as a total number of mapped isomiRs. The analysis of the differential expression of microRNAs between control and experimental samples were performed with DESeq2^40^.

### Affymetrix whole transcriptome gene expression analysis

Total RNA was isolated simultaneously with sRNA from Jurkat cells, and hybridized to a gene expression array (GeneChip Gene 1.0 ST Array System; Affymetrix, Santa Clara, CA). This array includes 28,869 well-annotated genes (from RefSeq, Ensembl and GenBank). Standard Affymetrix quality control was conducted using Expression Console^®^ Software. Genes were filtered by intensity compared with the control channel, and P ≤ 0.05 of a paired Student’s t-test was considered significant (ArrayTools was used for differential gene expression). For interpreting genome-wide expression profiles Gene Set Enrichment Analysis (GSEA) was applied. Microarray data are available in the ArrayExpress database (www.ebi.ac.uk/arrayexpress) under accession number E-MTAB-5574.

## Additional Information

**How to cite this article**: Mesitov, M. V. *et al*. Differential processing of small RNAs during endoplasmic reticulum stress. *Sci. Rep.* **7**, 46080; doi: 10.1038/srep46080 (2017).

**Publisher's note:** Springer Nature remains neutral with regard to jurisdictional claims in published maps and institutional affiliations.

## Supplementary Material

Supplementary Information 1

Supplementary Information 2
