# Supplementary material for: Differential processing of small RNAs during endoplasmic reticulum stress
Source: Sci Rep. 2017 Apr 13;7:46080. doi: 10.1038/srep46080 (PMC5408347; doi:10.1038/srep46080)
Supplement: Supplementary Information 1 [file srep46080-s1.pdf]

Differential processing of small RNAs during endoplasmic reticulum stress

Mikhail V. Mesitov[1,¶], Ruslan A. Soldatov[2,3,¶], Danila M. Zaichenko[1], Sophie G. Malakho[4], Tatyana S. Klementyeva[1], Alisa A. Sokolovskaya[1], Aslan A. Kubatiev[1,5], Andrey A. Mironov[2,3], Aleksey A. Moskovtsev*[1,5]

Table S1. Sequences of the PCR primers

Gene[a]	Accession number	Forward primer (5'–3')	Reverse Primer (5'–3')
BiP	NM_005347.4	CGAGGAGGAGGACAAGAAGG	CACCTTGAACGGCAAGAACT
XBP1(S)	NM_001079539.1	GGTCTGCTGAGTCCGCAGCAGG	GGGCTTGGTATATATGTGG
CHOP	NM_004083.5	TCAGAGCTGGAACCTGAGGAG	TTGGATCAGTCTGGAAAAGCA
ANG	NM_001145	CCTCCATGCCAGTACCGAG	GGACGACGGAAAATTGACTGA
RNH1	NM_203388	GAGCTGAGCGACGCTAGATG	GGGTTGACTCGAAGTGCAGAG
DROSHA	NM_013235.4	CCATCTCTGGAAAGGTCCTACA	CAGCAGGTTCAGGAACAACC
DICER1	NM_177438.2	GTCCGATGGTTCTCGAAGG	GCAAAGCAGGGCTTTTCA
AGO1	NM_012199.4	GACATCCCTAAGATCGACGTGT	CCACTTCCCGGTTGACTCTAC
AGO2	NM_012154.3	TCCACCTAGACCCGACTTTGG	GTGTTCCACGATTTCCCTGTT
AGO3	NM_024852.3	TGTCTCTCGGGTGAGTTGG	GAGAAAAATGAACGCCCCAC
AGO4	NM_017629.3	GGGTAGGGAAAAGTGGCAAT	AGCAGTTGTCATCCCACAAG
GAPDH	NM_002046.4	GAAGGTGAAGGTCGGAGTC	GAAGATGGTGATGGGATTTC

Table S2. Upregulated gene sets in DTT-treated Jurkat cells significant at FDR < 25%

#	NAME	SIZE	NOM p-val	FDR q-val
1	REACTOME PACKAGING OF TELOMERE ENDS	39	0.0000	0.0000
2	REACTOME DEPOSITION OF NEW CENPA CONTAINING NUCLEOSOMES AT THE CENTROMERE	48	0.0000	0.0000
3	KEGG SYSTEMIC LUPUS ERYTHEMATOSUS	107	0.0000	0.0000
4	REACTOME RNA POL I PROMOTER OPENING	45	0.0000	0.0000
5	REACTOME AMYLOIDS	63	0.0000	0.0000
6	REACTOME MEIOTIC RECOMBINATION	65	0.0000	0.0000
7	REACTOME RNA POL I TRANSCRIPTION	66	0.0000	0.0000
8	REACTOME MEIOTIC SYNAPSIS	59	0.0000	0.0000
9	REACTOME MEIOSIS	90	0.0000	0.0001
10	REACTOME TELOMERE MAINTENANCE	65	0.0000	0.0001
11	REACTOME RNA POL I RNA POL III AND MITOCHONDRIAL TRANSCRIPTION	95	0.0000	0.0001
12	REACTOME UNFOLDED PROTEIN RESPONSE	71	0.0000	0.0002
13	REACTOME DIABETES PATHWAYS	114	0.0000	0.0004
14	REACTOME CHROMOSOME MAINTENANCE	100	0.0000	0.0022
15	REACTOME ANTIGEN PRESENTATION FOLDING ASSEMBLY AND PEPTIDE LOADING OF CLASS I MHC	17	0.0000	0.0032
16	REACTOME ACTIVATION OF CHAPERONE GENES BY XBP1S	41	0.0015	0.0055
17	REACTOME AMINO ACID SYNTHESIS AND INTERCONVERSION TRANSAMINATION	16	0.0000	0.0077
18	REACTOME PERK REGULATED GENE EXPRESSION	25	0.0000	0.0078
19	REACTOME ACTIVATION OF GENES BY ATF4	22	0.0080	0.0372
20	REACTOME CYTOSOLIC TRNA AMINOACYLATION	22	0.0048	0.0426
21	KEGG P53 SIGNALING PATHWAY	65	0.0000	0.0505
22	REACTOME ASPARAGINE N LINKED GLYCOSYLATION	74	0.0014	0.0618
23	KEGG PROTEIN EXPORT	21	0.0031	0.0594
24	KEGG GLYCINE SERINE AND THREONINE METABOLISM	29	0.0031	0.0616
25	KEGG PANTOTHENATE AND COA BIOSYNTHESIS	16	0.0102	0.0672
26	ST TUMOR NECROSIS FACTOR PATHWAY	27	0.0079	0.0902
27	PID P38 ALPHA BETA DOWNSTREAM PATHWAY	36	0.0060	0.0950
28	REACTOME BIOSYNTHESIS OF THE N GLYCAN PRECURSOR DOLICHOL LIPID LINKED OLIGOSACCHARIDE LLO AND TRANSFER TO A NASCENT PROTEIN	18	0.0084	0.1376
29	BIOCARTA ATR BRCA PATHWAY	18	0.0084	0.1376
30	REACTOME INTERFERON GAMMA SIGNALING	53	0.0030	0.1401
31	REACTOME AMINO ACID TRANSPORT ACROSS THE PLASMA MEMBRANE	29	0.0178	0.1699
32	REACTOME SYNTHESIS OF PC	15	0.0261	0.1774
33	PID IL1 PATHWAY	27	0.0143	0.2015
34	KEGG SNARE INTERACTIONS IN VESICULAR TRANSPORT	33	0.0201	0.2119

Table S3. Downregulated gene sets in DTT-treated Jurkat cells significant at FDR < 25%

#	NAME	SIZE	NOM p-val	FDR q-val
1	REACTOME MRNA SPLICING	91	0.0000	0.0000
2	REACTOME PROCESSING OF CAPPED INTRON CONTAINING PRE MRNA	118	0.0000	0.0000
3	KEGG TASTE TRANSDUCTION	49	0.0000	0.0015
4	REACTOME MRNA PROCESSING	135	0.0000	0.0110
5	KEGG SPLICEOSOME	102	0.0000	0.0928
6	REACTOME REGULATORY RNA PATHWAYS	22	0.0053	0.0940
7	PID NOTCH PATHWAY	54	0.0000	0.0953
8	REACTOME MICRORNA MIRNA BIOGENESIS	20	0.0000	0.0955
9	REACTOME FORMATION OF TUBULIN FOLDING INTERMEDIATES BY CCT TRIC	17	0.0052	0.1130
10	REACTOME TRANSPORT OF MATURE TRANSCRIPT TO CYTOPLASM	42	0.0031	0.1885
11	BIOCARTA TEL PATHWAY	16	0.0076	0.2065
12	REACTOME OLFACTORY SIGNALING PATHWAY	285	0.0000	0.2159
13	REACTOME TRANSPORT OF MATURE MRNA DERIVED FROM AN INTRONLESS TRANSCRIPT	31	0.0054	0.2190
14	REACTOME MRNA 3 END PROCESSING	25	0.0053	0.2242
15	REACTOME INTEGRIN ALPHAIIB BETA3 SIGNALING	26	0.0085	0.2323

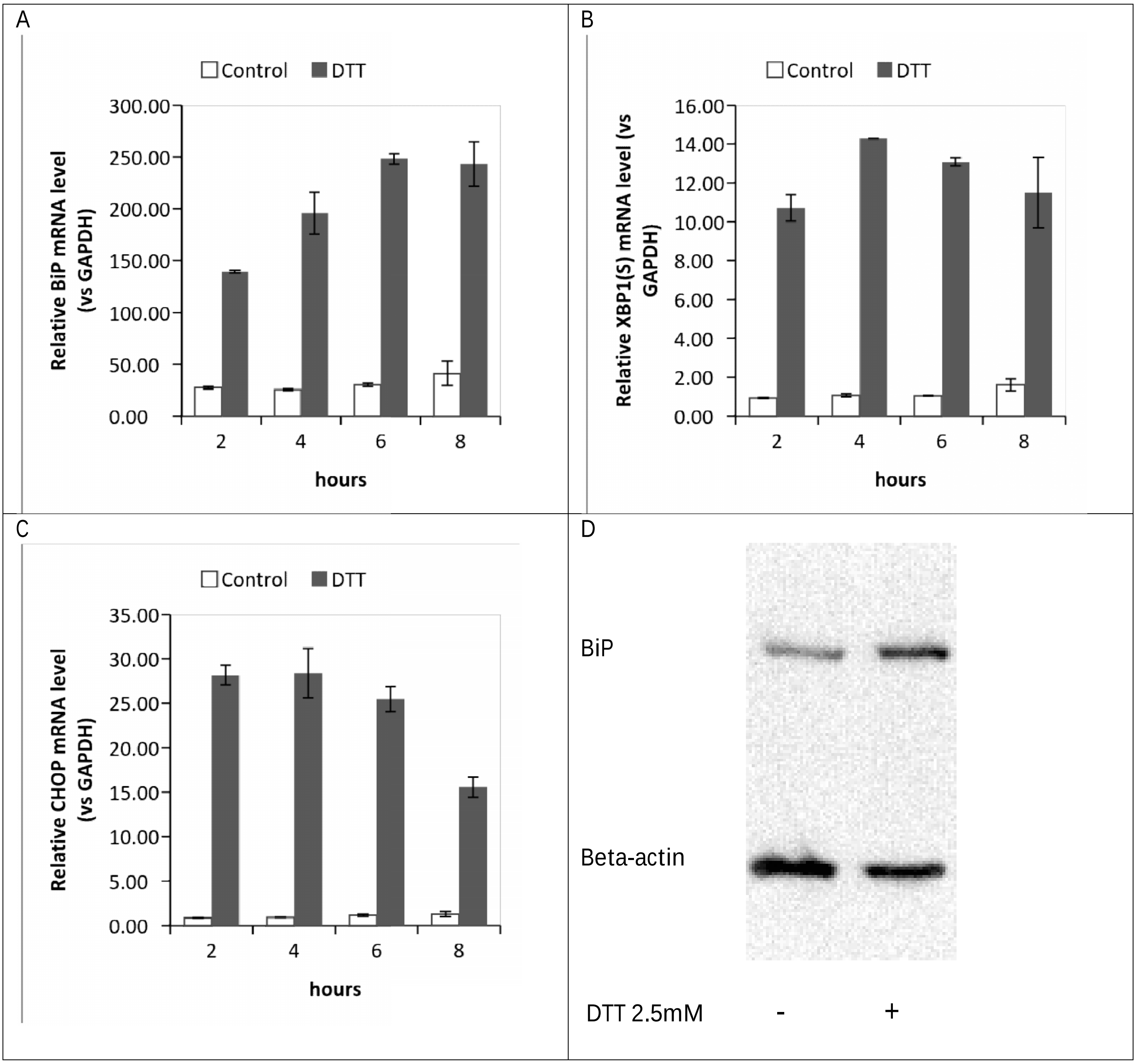

Figure S1. Expression levels of (A) BiP, (B) XBP1(S), (C) CHOP mRNAs and BiP protein (D) in Jurkat cells under ER stress. Data in A, B, C are presented as the mean ± SD, differences between mRNA expression in control and DTT-treated samples are significant ($p<0.05$) at each time point for all genes. The p-value was calculated versus control by two-tailed Student's t test. For A, B, C, D n=3

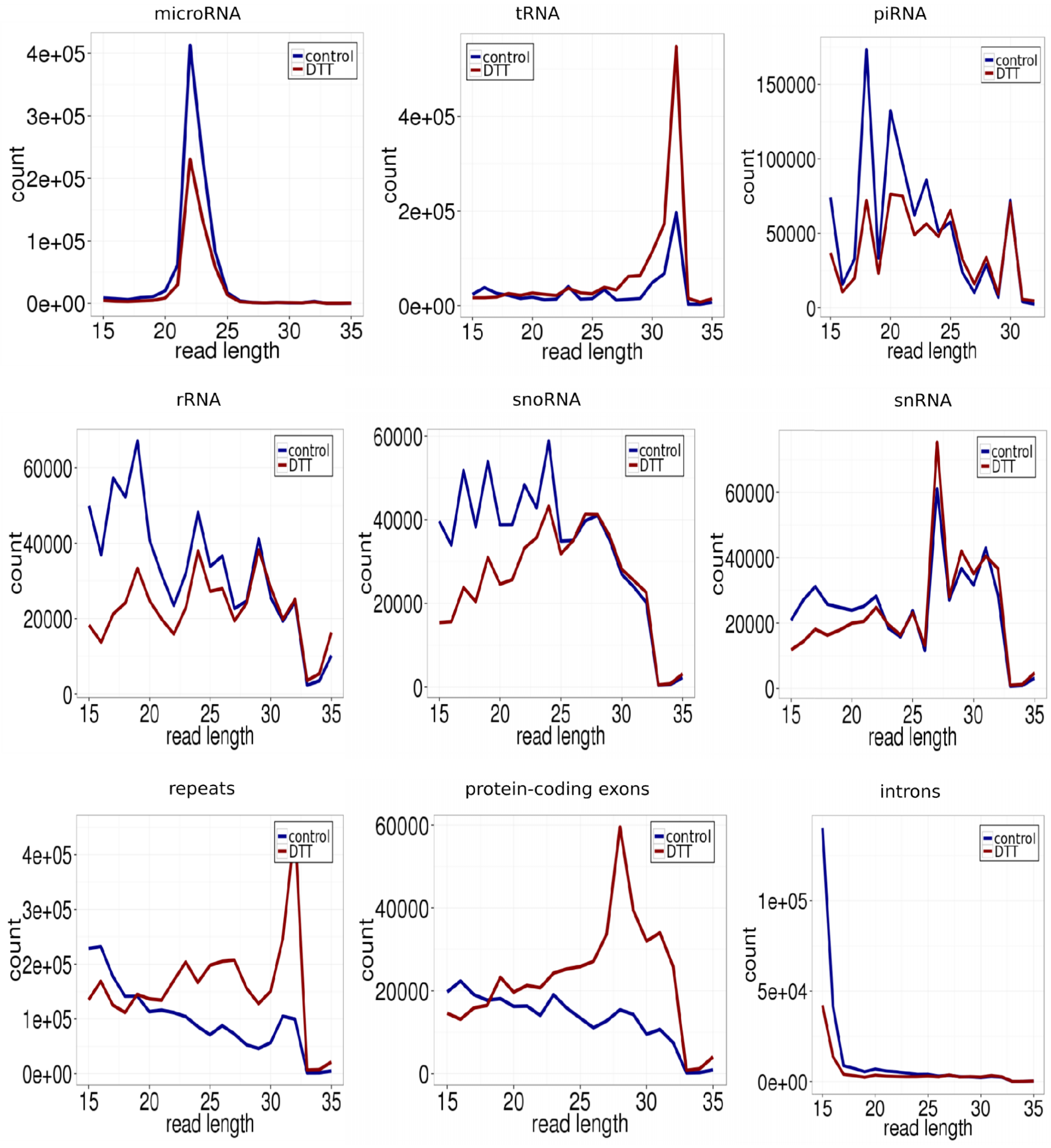

Figure S2. Distribution of sequenced reads mapped without mismatches on different RNA classes in ER-stressed and control Jurkat T-cells.

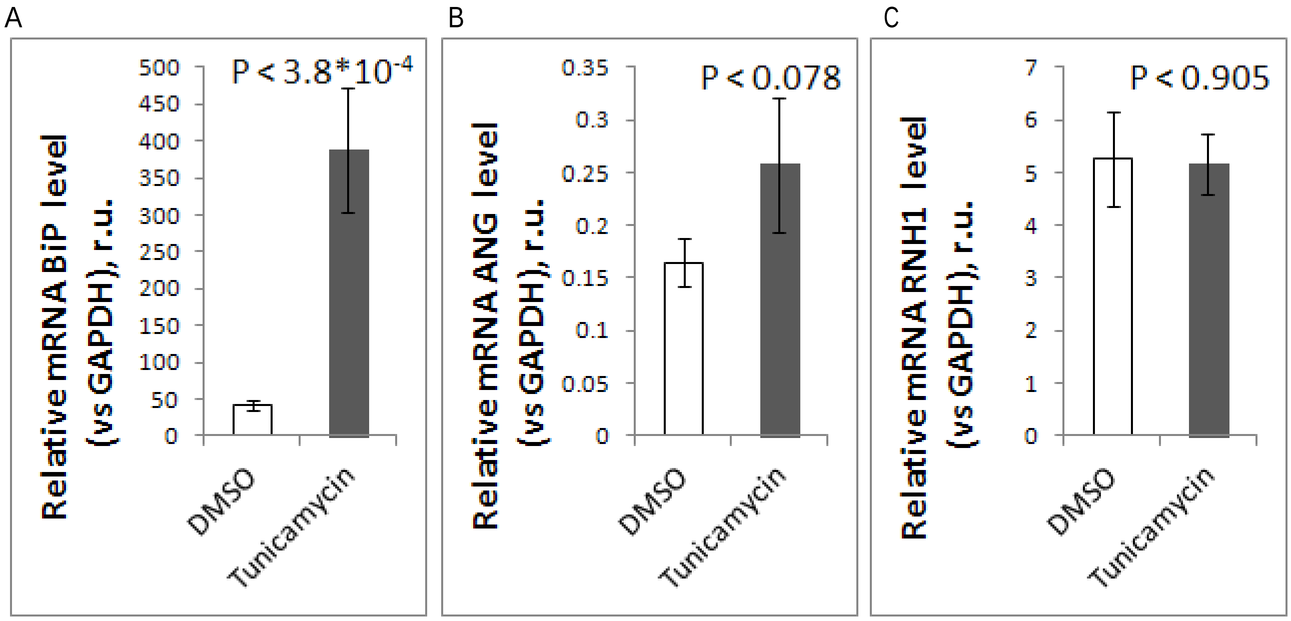

Figure S3. Expression levels of (A) BiP, (B) ANG, (C) RNH1 mRNAs in Jurkat cells under tunicamycin 10 mkg/ml treatment, 6h . Data in a,b,c are presented as the mean ± SD (n=3). The p-value was calculated versus control by two-tailed Student's t test.

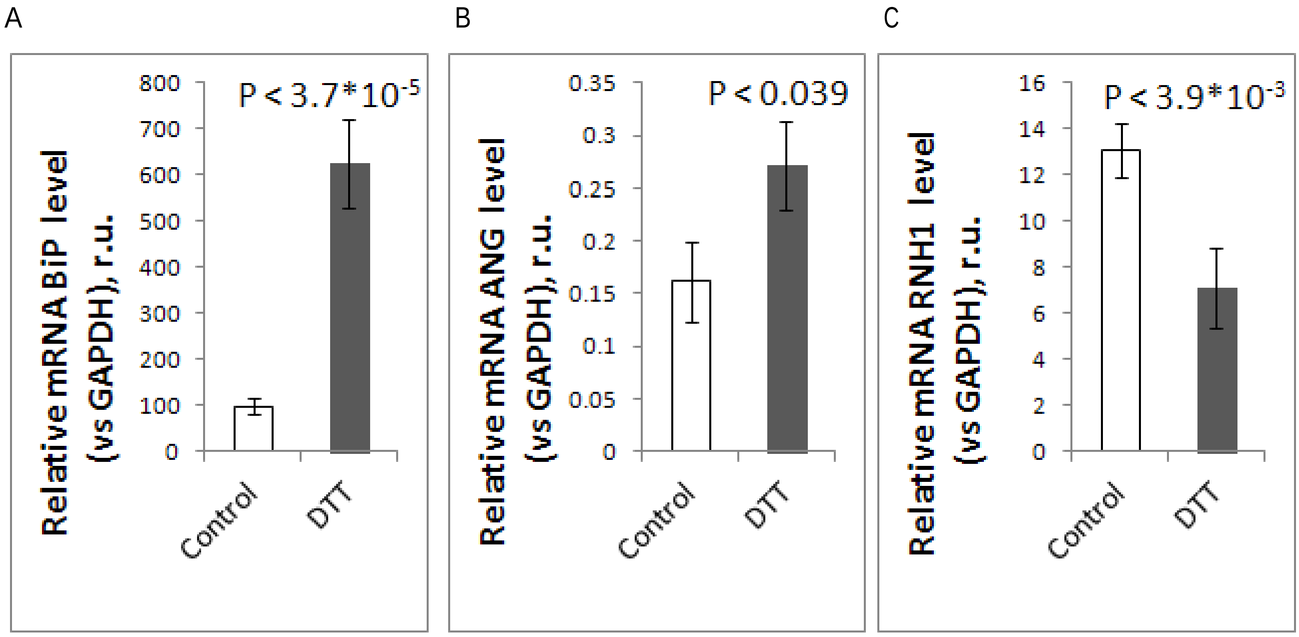

Figure S4. Expression levels of (A) BiP, (B) ANG, (C) RNH1 mRNAs in EA.hy926 cells under DTT 2.5 mM treatment, 6h . Data in a,b,c are presented as the mean ± SD (n=3). The p-value was calculated versus control by two-tailed Student's t test.

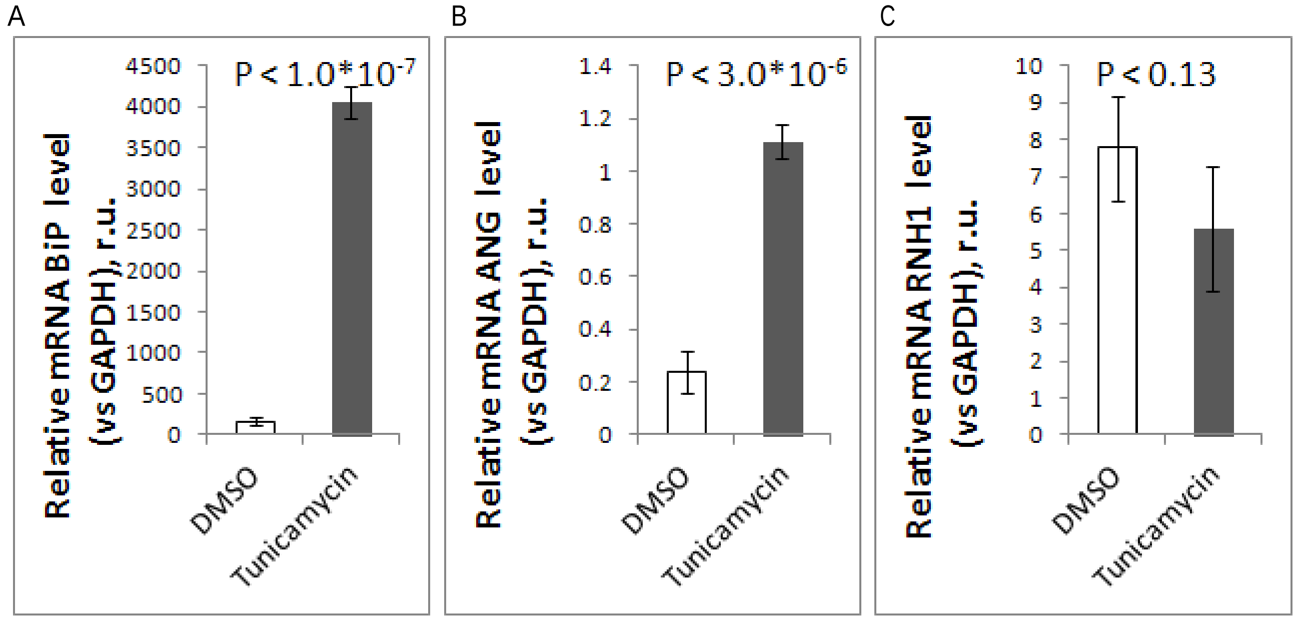

Figure S5. Expression levels of (A) BiP, (B) ANG, (C) RNH1 mRNAs in EA.hy926 cells under tunicamycin 10 mkg/ml treatment, 6h . Data in a,b,c are presented as the mean ± SD (n=3). The p-value was calculated versus control by two-tailed Student's t test.

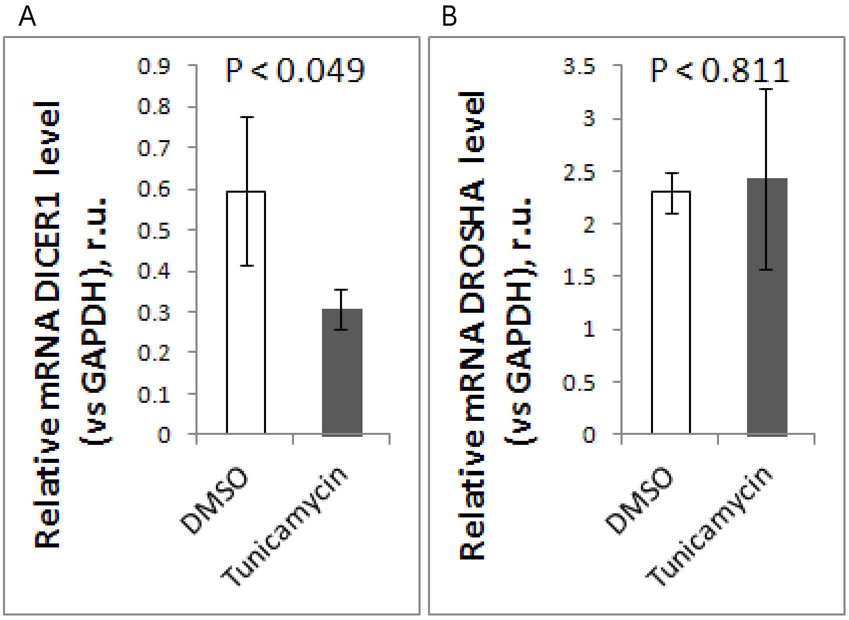

Figure S6. Expression levels of (A) DICER1, (B) DROSHA mRNAs in Jurkat cells under tunicamycin 10 mkg/ml treatment, 6h . Data in a,b,c are presented as the mean ± SD (n=3). The p-value was calculated versus control by two-tailed Student's t test.

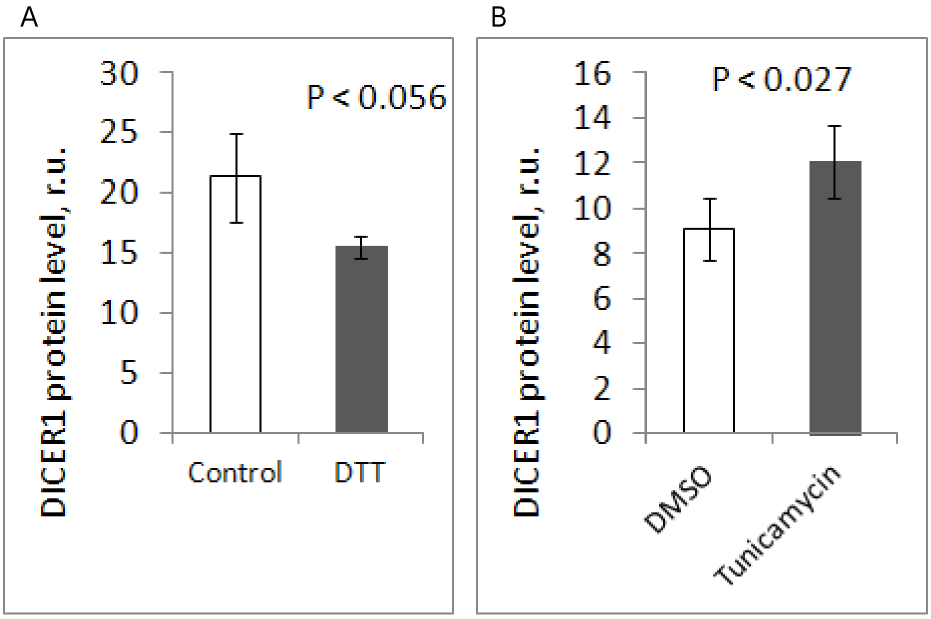

Figure S7. Expression levels of DICER1 protein in Jurkat cells under (A) DTT 2.5mM and (B) tunicamycin 10 mkg/ml treatment, 6h. Data in a,b are presented as the mean ± SD (n=3). The p-value was calculated versus control by two-tailed Student's t test. Please note, that DMSO treatment alone leads to significant decrease in DICER1 protein level approximately two-fold in Jurkat cells.

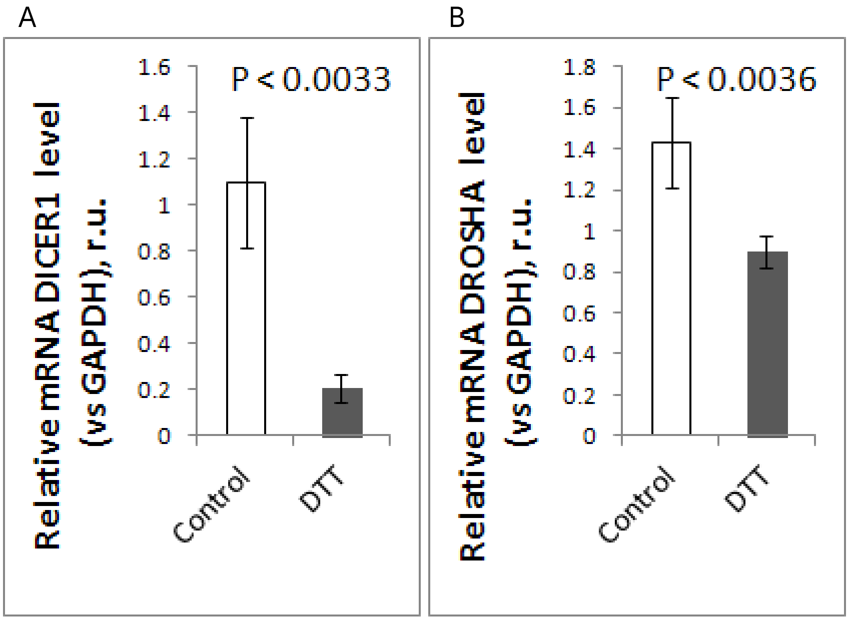

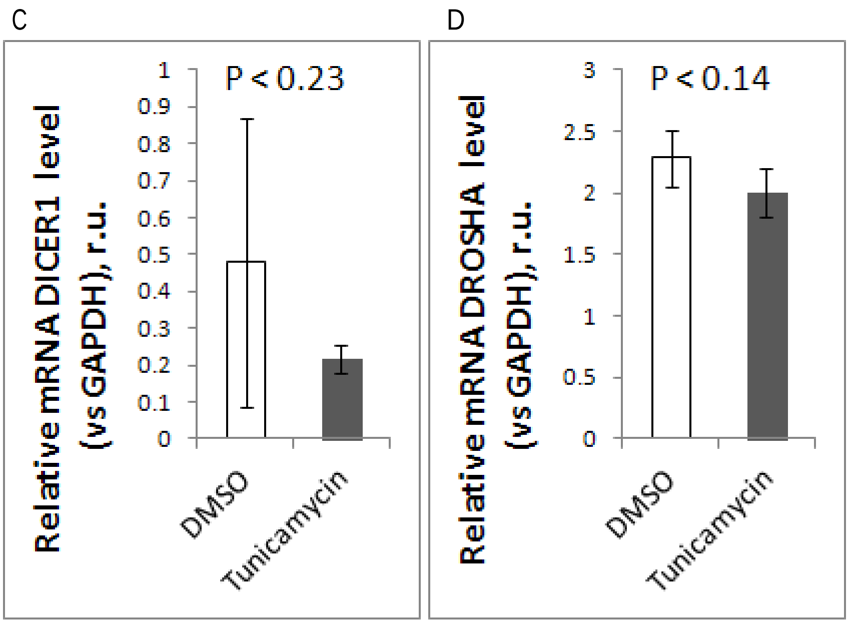

Figure S8. Expression levels of (A), (C) DICER1, (B),(D) DROSHA mRNAs in EA.hy 926 cells under DTT 2.5 mM (A,B) and tunicamycin (C,D) 10 mkg/ml treatment, 6h . Data in a,b,c,d are presented as the mean ± SD (n=3). The p-value was calculated versus control by two-tailed Student's t test.

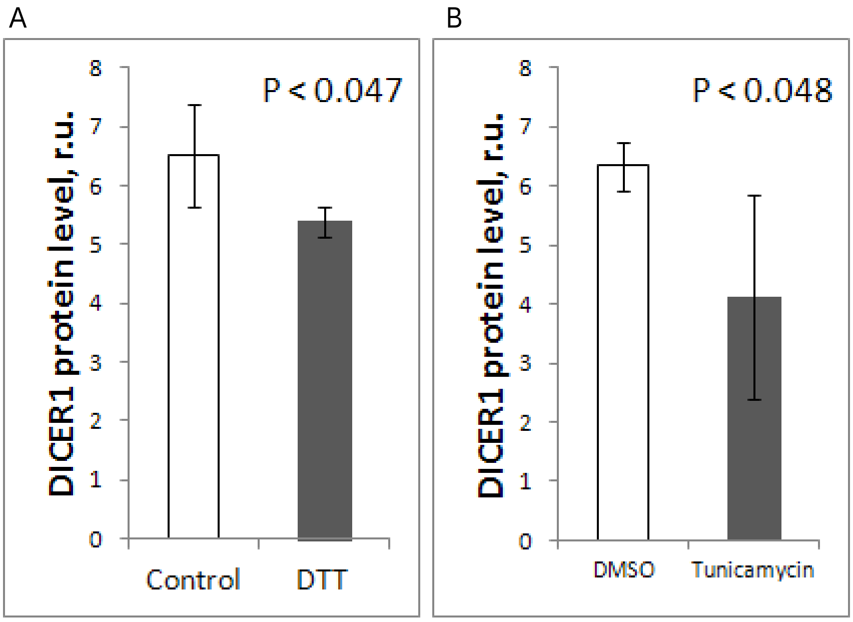

Figure S9. Expression levels of DICER1 protein in EA.hy926 cells under (A) DTT 2.5mM and (B) tunicamycin 10 mkg/ml treatment, 6h . Data in a,b are presented as the mean ± SD (n=3). The p-value was calculated versus control by two-tailed Student's t test.

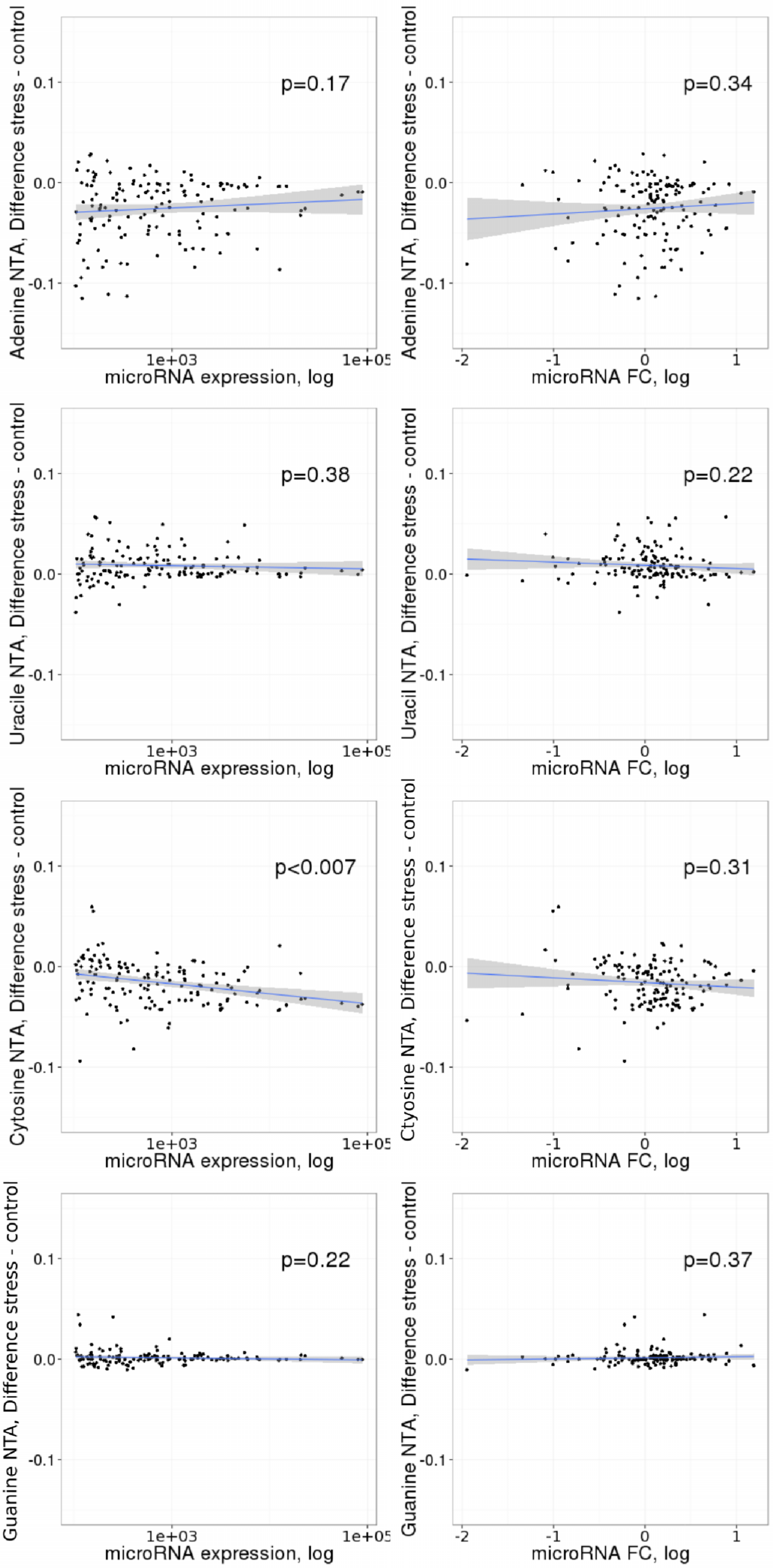
Adenine NTA, Difference stress - control
p=0.17
p=0.34
Uracile NTA, Difference stress - control
Uracil NTA, Difference stress - control
p=0.38
p=0.22
Cytosine NTA, Difference stress - control
Ctyosine NTA, Difference stress - control
p<0.007
p=0.31
Guanine NTA, Difference stress - control
p=0.22
p=0.37
0.1
0.0
-0.1
1e+03
1e+05
-2
-1
0
1
microRNA expression, log
microRNA FC, log

Figure S10. Correlation between microRNA expression and variation in NTA fraction (left figures); microRNA fold change and variation in NTA fraction (right figures).

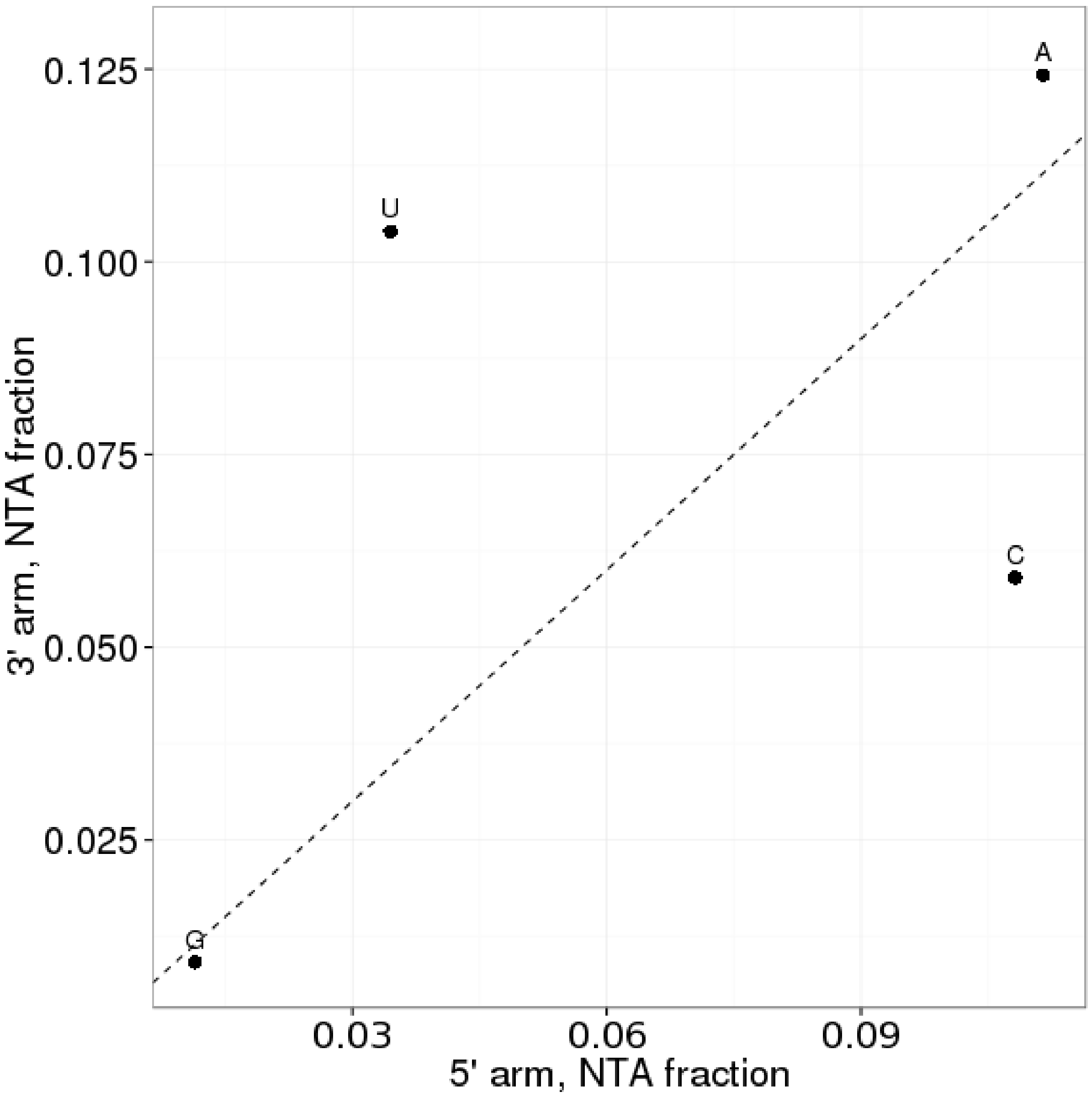

Figure S11. Fraction of 3’NTAs separately among 5’ arm and 3’ arm of microRNAs. Dashed line corresponds to y=x.

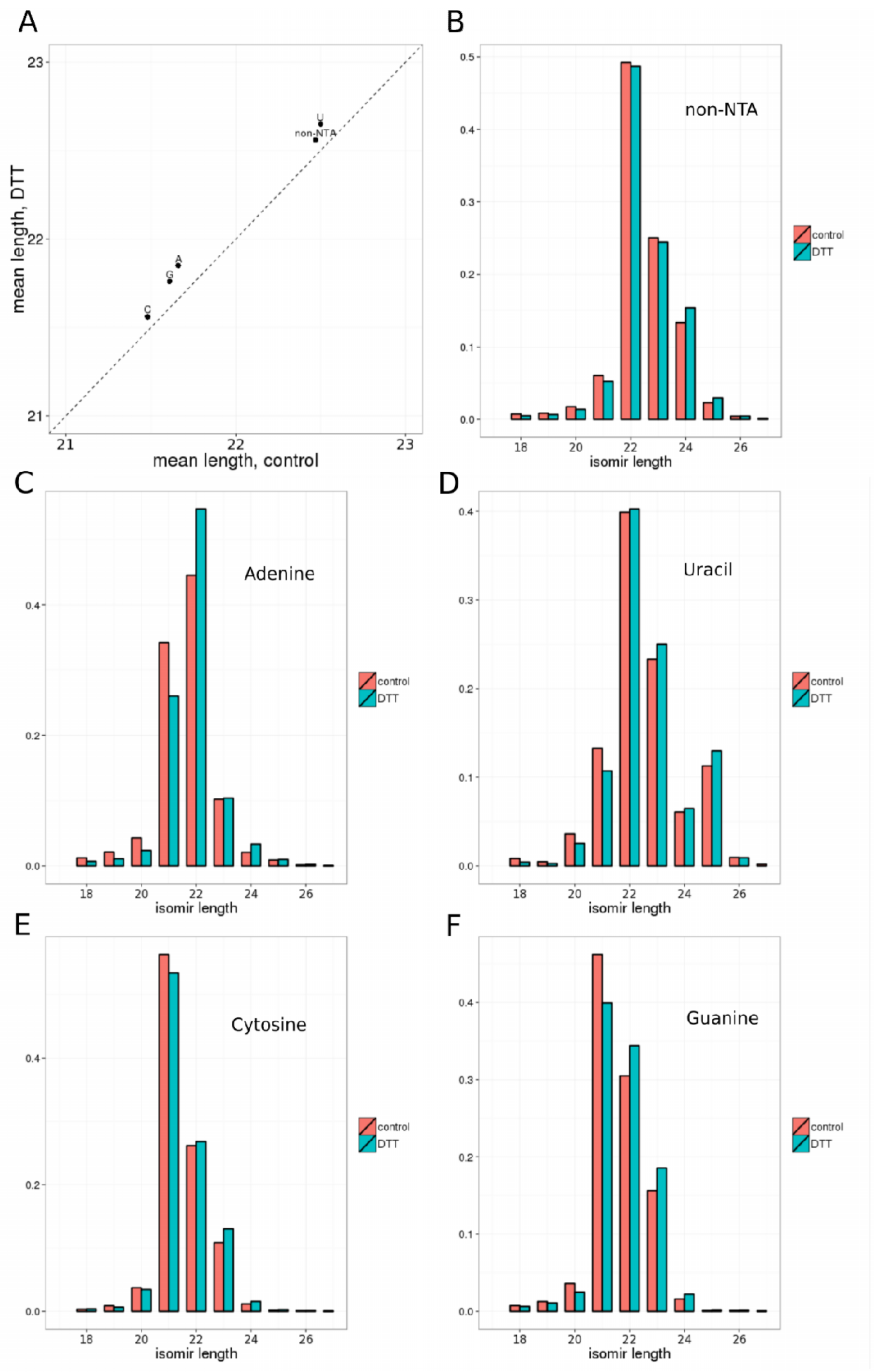

Figure S12. MiRNA length in control and ER-stressed Jurkat T-cells. A) Mean length of different types of miRNA isoforms. Dashed line corresponds to y=x. B)-F) Distribution of miRNA isoform length for non-NTA isomirs (B) and pre-modified NTA-A (C), NTA-U (D), NTA-C (E), NTA-G (F).

A

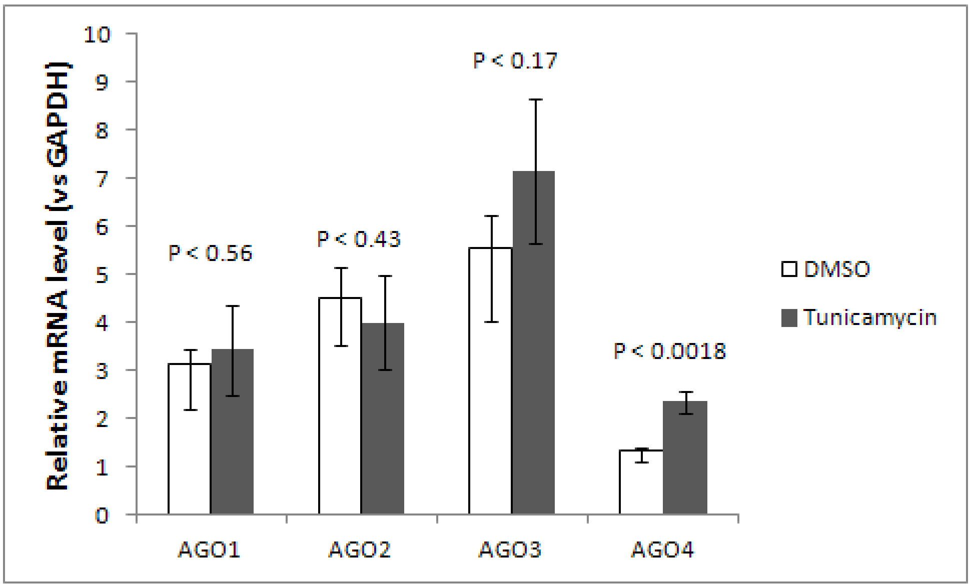

Relative mRNA level (vs GAPDH)
10
9
8
7
6
5
4
3
2
1
0
P < 0.56
P < 0.43
P < 0.17
P < 0.0018
AGO1
AGO2
AGO3
AGO4
DMSO
Tunicamycin

B

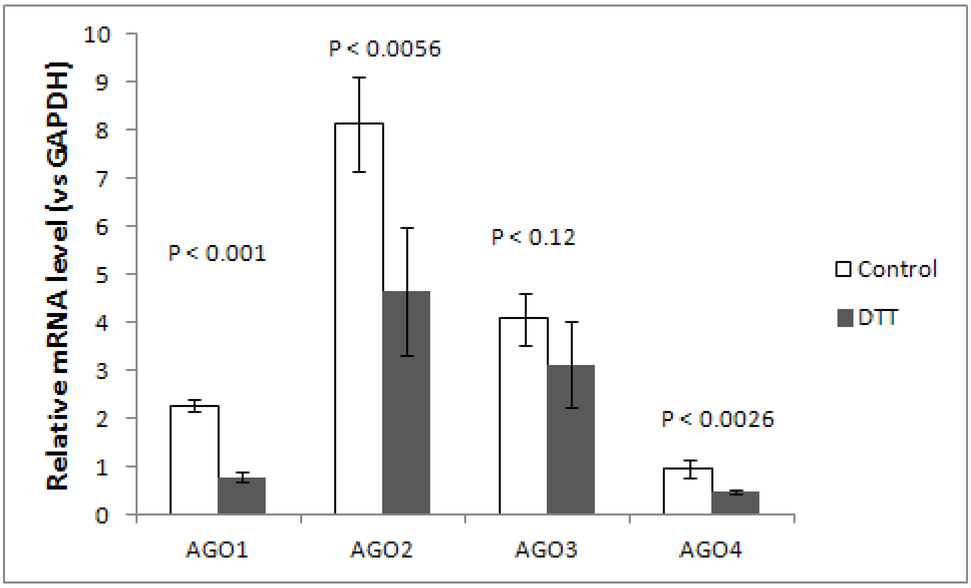

Relative mRNA level (vs GAPDH)
10
9
8
7
6
5
4
3
2
1
0
P < 0.001
P < 0.0056
P < 0.12
P < 0.0026
AGO1
AGO2
AGO3
AGO4
Control
DTT

C

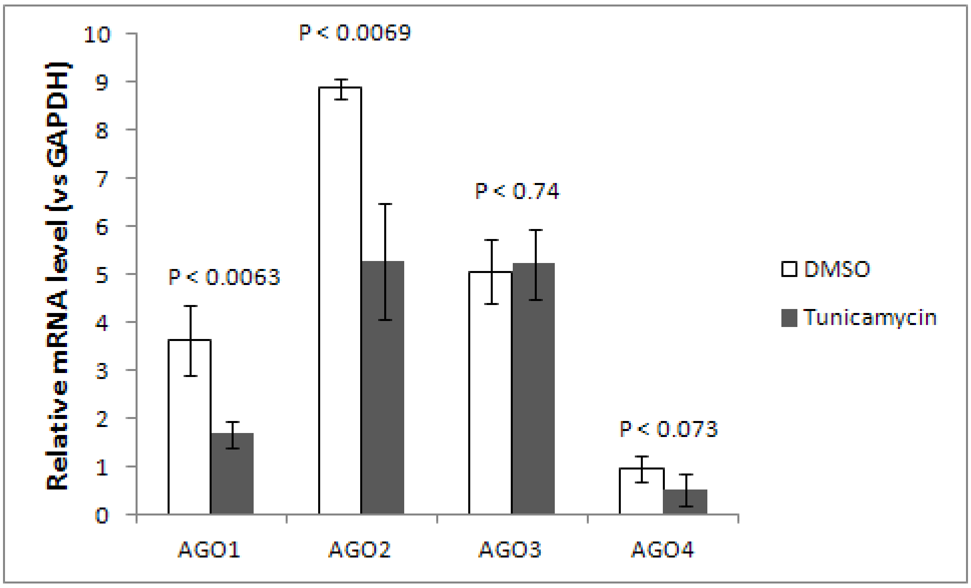

Relative mRNA level (vs GAPDH)
10
9
8
7
6
5
4
3
2
1
0
P < 0.0063
P < 0.0069
P < 0.74
P < 0.073
AGO1
AGO2
AGO3
AGO4
DMSO
Tunicamycin

Figure S13. Expression levels of AGO1, AGO2, AGO3, AGO4 in Jurkat (A) and EA.hy926 cells (B,C) under (B) DTT 2.5mM and (A,C) tunicamycin 10 mkg/ml treatment, 6h . Data in a,b,c are presented as the mean ± SD (n=3). The p-value was calculated versus control by two-tailed Student's t test.
